# Genome elimination from the germline cells in diploid and triploid male water frogs *Pelophylax esculentus*

**DOI:** 10.3389/fcell.2022.1008506

**Published:** 2022-10-14

**Authors:** Magdalena Chmielewska, Mikołaj Kaźmierczak, Beata Rozenblut-Kościsty, Krzysztof Kolenda, Anna Dudzik, Dmitrij Dedukh, Maria Ogielska

**Affiliations:** ^1^ Amphibian Biology Group, Department of Evolutionary Biology and Conservation of Vertebrates, Faculty of Biological Sciences, University of Wrocław, Wrocław, Poland; ^2^ Department of Medicine Biology, The Cardinal Wyszyński National Institute of Cardiology, Warsaw, Poland; ^3^ Laboratory of Non-Mendelian Evolution, Institute of Animal Physiology and Genetics, Czech Academy of Sciences, Liběchov, Czech Republic

**Keywords:** hybridogenesis, *Pelophylax esculentus*, spermatogenesis, genome elimination, endoreplication, polyploidy, aneuploidy, *in situ* hybridization

## Abstract

Hybridogenesis is a hemiclonal reproductive strategy in diploid and triploid hybrids. Our study model is a frog *P. esculentus* (diploid RL and triploids RLL and RRL), a natural hybrid between *P. lessonae* (LL) and *P. ridibundus* (RR). Hybridogenesis relies on elimination of one genome (L or R) from gonocytes (G) in tadpole gonads during prespermatogenesis, but not from spermatogonial stem cells (SSCs) in adults. Here we provide the first comprehensive study of testis morphology combined with chromosome composition in the full spectrum of spermatogenic cells. Using genomic *in situ* hybridization (GISH) and FISH we determined genomes in metaphase plates and interphase nuclei in Gs and SSCs. We traced genomic composition of SSCs, spermatocytes and spermatozoa in individual adult males that were crossed with females of the parental species and gave progeny. Degenerating gonocytes (24%–39%) and SSCs (18%–20%) led to partial sterility of juvenile and adult gonads. We conclude that elimination and endoreplication not properly completed during prespermatogenesis may be halted when gonocytes become dormant in juveniles. After resumption of mitotic divisions by SSCs in adults, these 20% of cells with successful genome elimination and endoreplication continue spermatogenesis, while in about 80% spermatogenesis is deficient. Majority of abnormal cells are eliminated by cell death, however some of them give rise to aneuploid spermatocytes and spermatozoa which shows that hybridogenesis is a wasteful process.

## Introduction

Hybridogenesis is one of the modifications of gametogenesis that allows reproduction of some interspecific diploid and polyploid hybrids (Dawley and Bogart, 1989). It relies on elimination of one of the parental genomes from germ line cells of a hybrid and transmission of another one to the gametes. The elimination is usually followed by duplication of the genome that remains, which in turn allows meiosis to occur. However, the resulting gametes are clonal because crossing-over that occurs between genetically identical reduplicated chromatids does not result in recombination. For this reason, hybridogenesis is often defined as asexual reproduction [for review see (Lamatsch and Stöck, 2009)].

This peculiar process has emerged in animals independently in various animal taxa including insects, fish and amphibians (Schultz, 1969; Berger, 1973; Scali et al., 2003; Plötner, 2005; Ogielska et al., 2010; Stöck et al., 2012; Majtánová et al., 2021). The European water frog complex of the genus *Pelophylax,* which is the subject of this study, is composed of two diploid species: *P. lessonae* (LL) and *P. ridibundus* (RR), where complete 2n chromosomal set consists of 26 chromosomes, and their interspecific hybrids *P. esculentus* (E) represented by diploid (RL, 2n = 26 chromosomes) and triploid (RRL and LLR, 3n = 39 chromosomes) individuals of both sexes. The hybrids usually coexist in the same population with one or both of the parental species (R-E and L-E or R-E-L systems, respectively), or in populations composed of diploid and triploid hybrids (E-E system) (Rybacki and Berger, 2001; Plötner, 2005; Christiansen, 2009; Dufresnes and Mazepa, 2020). To propagate themselves over generations, hybrids must produce either R or L gametes and mate with this parental species, whose genome was eliminated or with another hybrid that has eliminated the opposite genome. Triploid hybrids emerge in rare cases when hybrid individuals produce diploid gametes (RL, LL or RR) (Uzzell, Berger and Günther, 1975; Berger, Roguski and Uzzell, 1978; Christiansen, 2009; Pruvost, Hoffmann and Reyer, 2013; Dedukh et al., 2017; Dedukh et al., 2022a). On the other hand, triploids produce haploid gametes and transmit to progeny the genome which is double-copied (e.g., RRL produces R gametes) (Christiansen, 2009; Dedukh et al., 2015; Dedukh et al., 2022b). A commonly accepted model of hybridogenesis in water frogs assumes that the genome that remains reduplicates before meiotic recombination and forms identical (clonal) copies that eventually are transmitted to clonal gametes. In *P. esculentus*, the process was described in oogenesis (Graf and Müller, 1979; Bucci et al., 1990; Dedukh et al., 2015) and was first reported by Tunner and Heppich (Tunner and Heppich, 1981) and Tunner and Heppich-Tunner (Tunner and Heppich-Tunner, 1991) in juvenile hybrid females.

Hybridogenetic gametogenesis usually occurs in females, except of diploid fish *Hypseleotris spp* (Schmidt et al., 2011; Majtánová et al., 2021) and triploid green toad *Bufotes pseudoraddei baturae* (Stöck et al., 2002, 2012) where both sexes eliminate one of the genomes. The same process evidently also occurs in male *P. esculentus*, although it was proven in most cases indirectly by analysis of genome composition in progeny resulting from experimental crosses between parental species and hybrids, as evidenced by classic studies of Berger (Berger, 1968, 1973, 1976) and others (Dedukh et al., 2017; Dedukh et al., 20220b; Dedukh et al., 2022b). The other evidence comes from studies on genome compositions of spermatozoa detected by flow cytometry that allowed distinguishing genomes on the basis of species-specific difference in amount of DNA in *P. lessonae* and *P. ridibundus* (Mazin and Borkin, 1979; Vinogradov et al., 1990; Biriuk et al., 2016). Direct evidences of genome elimination in spermatogenic cells in males are not numerous and restricted to very limited number of juvenile and adult individuals (Heppich, Tunner and Greilhuber, 1982; Ragghianti et al., 2007; Doležálková et al., 2016). To understand these difficulties, we must go back to the basics of the process of spermatogenesis and focus on germ cells called “primary spermatogonia”. In a recent paper, Haczkiewicz, Rozenblut-Kościsty and Ogielska (2017) presented evidence of existence of two classes of “primary spermatogonia” in the parental species *P. lessonae* and *P. ridibundus*: [1] gonocytes (G) present only during prespermatogenesis, i.e., restricted to the larval and tadpole stages before the completion of metamorphosis; G are descendants of embryonic primordial germ cells (PGCs) after their migration into an early gonad where - after several mitotic cycles - become dormant until sexual maturity when they transform into [2] spermatogonial stem cells (SSCs) present during active spermatogenesis in adult males. Only SSCs proliferate throughout the whole adult life of a male giving rise to meiocytes and spermatozoa and renovating the pool of SSCs. The distinction between G and SSC was not taken into account in studies on spermatogenesis in amphibians in general, and in hybridogenetic water frogs in particular.

Spermatogenesis—in contrast to oogenesis—enables studies on all stages of meiosis from the very beginning (G and formation of the renewable pool of SSCs—see: Haczkiewicz et al., 2017) through formation of cysts with proliferating secondary spermatogonia that enter prophase of the first meiotic division (primary spermatocytes: leptotene, zygotene, pachytene, diplotene and diakinesis), metaphases of the first and second meiotic divisions and, finally, formation of spermatids and spermatozoa that are released from cysts during spermiation (Ogielska and Bartmańska, 2009; Rozenblut-Kościsty et al., 2017; Roco, Ruiz-García and Bullejos, 2021).

In this study we made an assumption that the two classes of “primary spermatogonia”, namely G and SSC, in hybrid frogs are not equal in their ability to genome elimination. We argue that only G (but not SSC) are competent in chromosome rejection. The elimination takes place during interphase *via* micronuclei budding off from gonocyte nuclei (Ogielska, 1994; Chmielewska et al., 2018) and, probably, in the course of mitosis *via* micronuclei arising from chromosomes lagging at anaphase (Ogielska, 1994; Dedukh et al., 2019; Dedukh et al., 2022b). These small chromatin bodies contain the rejected chromosomes (Dedukh et al., 2017; Dedukh et al., 2019; Chmielewska et al., 2018; Dedukh et al., 2020b).

We tested the hypothesis that elimination of chromosomes occurs only in gonocytes (G) during prespermatogenesis in tadpoles. We assumed that PGCs and G initially have the same chromosome composition as somatic cells (RL in diploids and RRL or RLL in triploids), gonocytes from sexually differentiated testes in tadpoles have variable compositions of L and R, while the resulting SSCs in adults contain chromosomes of only one of the parental species (RR or LL). Elimination does not continue in SSCs and their descendant germ cells (secondary spermatogonia, spermatocytes, spermatids and spermatozoa) in adult males. In case when elimination from G was correct (i.e., consistent with the generally accepted model), active spermatogenesis in hybrid males should be the same as in the parental species and spermatozoa should transmit only R or L genomes.

To check our hypothesis, we performed three approaches. First, we studied gonadal morphology and chromosome composition of the whole spectrum of male germ cells during ontogeny from undifferentiated gonads until sexually mature testes. Second, to identify ploidy level, we combined cytogenetic analysis of dividing PGCs, Gs and SSCs (AMD/DAPI, GISH and FISH) with morphology of spermatogenic cells on histological sections of the same gonad (in adults) or gonads in siblings (in tadpoles). Third, to identify genome contribution (L or R) into viable progeny, we performed classic crosses experiments of the hybridogenic males with LL and RR females as well as cytological and histological examination of testes from the same male. The combination of the mentioned approaches enabled us to analyze, at which moment of male ontogenesis the genome elimination occurs, and which of the genomes are transmitted to functional spermatozoa.

## Materials and methods

### Animals and crossings

Altogether 59 adult individuals were used in the study. The sites of capture, types of populations, GPS coordinates, morphological phenotypes, genotyping data and individuals’ age are listed in Supplementary Table S1. Altogether, we collected 23 *P. esculentus* individuals (4 females, 19 males), 17 *P. lessonae* individuals (5 females, 12 males) and 19 *P. ridibundus* individuals (7 females, 12 males).

We performed series of 35 *in vitro* crossings in 2014, 2015, and 2016 to obtain hybrids of different ploidy and genomic composition for histological, chromosomal and male fertility studies (Supplementary Figure S1, Supplementary Table S2). As parents we used: *P. esculentus* - 10 adult males (5 diploid RL, 3 RLL and 2 RRL triploids), and 4 diploid females from E-E population in N-W Poland (Wysoka Kamieńska) *P. lessonae*—12 males and 5 females, *P. ridibundus*—9 males and 7 females (Supplementary Table S2). To check the hybrid male fertility and genome transmission to the offspring, we selected 5 diploid and 5 triploid (3 RLL and 2 RRL) males and crossed them with *P. lessonae* (LL) or *P. ridibundus* (RR) females (Supplementary Table S11). The mothers produced eggs of predictable L or R genotypes, respectively, and their fertility was confirmed in control crosses (Supplementary Table S11).

Artificial crossing experiments were performed according to the standard procedures for water frogs (Berger et al., 1994). 24 h before the procedure, gravid females were injected intraperitoneally with salmon luteinizing hormone-releasing hormone (LHRH, H-7525.0001, Bachem) in amphibian PBS (APBS, pH 7.4, 11.2 mM NaCl, 0.22 mM KCl, 0.8 mM Na_2_HPO_4_, 0.14 mM KH_2_PO_4_) in amount of 6.25 mg/kg of body weight. When females started ovulation, the portions of oocytes were gently squeezed to plastic dishes and fertilized with spermatozoa by spreading the homogenate from male testes. After short anaesthesia (i.e., when a frog stops moving and looks faint) in 0.5% solution of ethyl 3-aminobenzoate methanesulfonate (MS-222, Sigma Chemical Co.) in APBS, the males’ body cavities were open by a small incision to dissect one of the testes (usually left) which was used for *in vitro* fertilization (Supplementary Figure S1A). The incision was sutured with a surgical thread (4-0 Dexon II) and the frog was rinsed and put on a wet tissue until awakened. After 2–3 weeks such males were injected intra-peritoneally with 1 ml of 0.3% aqueous solution of colchicine (Sigma Aldrich), and 24 h later they were fully anesthetized and sacrificed by intersection of the spinal cord. We dissected intestine for somatic genotype assessment by karyotyping and the remaining second testis for chromosome preparations and histology (Supplementary Figure S1B). Tadpoles resulting from *in vitro* crosses were reared in a greenhouse in plastic tanks according to Berger, Rybacki and Hotz (1994).

Altogether, 287 tadpoles were analyzed: 63 *P. ridibundus* tadpoles (progeny of 2 pairs, 2 *P. esculentus* males crossed with 2 *P. ridibundus* females), and 226 *P. esculentus* tadpoles (progeny of 25 pairs) (for details see Supplementary Table S3). *P. esculentus* tadpoles were obtained by crossing *P. lessonae* females with *P. ridibundus* males (6 crosses), *P. ridibundus* females with *P. lessonae* males (5 crosses), *P. esculentus* females with *P. lessonae* males (6 crosses of 3 females) or *P. esculentus* females with *P. ridibundus* males (3 crosses of 2 females) (Supplementary Figure S1C), and *P. esculentus* males (5 diploid RL and 5 triploid, 3 RLL and 2 RRL) with *P. lessonae* (2 crosses) or *P. ridibundus* females (4 crosses). 27 tadpoles were used for histology, another 36 tadpoles were used for gonadal chromosomal preparations, and 223 tadpoles were used for genotyping to assess the gametes genome composition of diploid and triploid hybrid fathers. Tadpoles were incubated overnight in a 0.01% solution of colchicine in tap water followed by anesthetizing in 0.25% solution of MS-222 in APBS. Afterwards, gonads for morphological and immunohistochemical analyses were dissected.

All procedures were carried out in accordance with existing Polish guidelines and legislation. The collection of all specimens was approved by the Polish General and Regional Directorates for Environmental Protection (DOPg 4201-02-74/05, DOP-oz.6401.02.2.2013.JRO, DOP-oz.6401.02.2.2013.2014.JRO.as, WPN.6205.28.2014.IW.2, DZP-WG.6401.02.5.2015.JRO, WPN.6401.177.2016.IL) and further laboratory activities were accepted by the Local Commission for Ethics in Experiments on Animals in Wrocław, Poland (103/2007, 7/2013, 27/2016).

### Taxonomic evaluation

Initial taxonomic evaluation of adult frogs was performed using morphological criteria and according to Kierzkowski et al. (2013). Ploidy of *P. esculentus* individuals was estimated using erythrocyte long axes measurements on air dried smears (Ogielska et al., 2005; Kierzkowski et al., 2011) using Axiostar Plus microscope (Zeiss) equipped with ×20 lens and KS400 software (Zeiss). Diploid erythrocytes were 23.4–24.9 µm long and triploid were 29.5–33.3 µm. Morphological evaluation of parental individuals as well as tadpoles from artificial crosses was further confirmed by one of the following methods: 1) AMD-DAPI analysis of metaphase plates and interphase cells of gut epithelium (Heppich et al., 1982; Ogielska et al., 2005), where pericentromeric heterochromatin of *P. ridibundus* chromosomes displays intensive DAPI (4′-6-diamidine-2-phenylindol, SIGMA) signals. 2) Allele size polymorphism in serum albumin introne-1 (Hauswaldt et al., 2012) with some modifications in PCR protocol (Kolenda et al., 2017). 3) We additionally used FISH with telomeric probe allowing detection of interstitial telomeric loci, differing in number in both parental species (according to Dedukh et al., 2013; Dedukh et al., 2015). Additionally, age of all adult males analyzed in chromosomal study was estimated by skeletochronology of the phalangeal bones (Rozenblut and Ogielska, 2005).

### Histology and immunohistochemistry

Dissected gonads were fixed in Bouin solution (Sigma-Aldrich, Germany), for 4–5 h (adults), and for 2–3 h (tadpoles). After washing with 70% ethanol, tissues were dehydrated in graded ethanol and xylene, embedded in paraplast, sectioned on Leica RM 2255 microtome into 7 µm-thick sections, stained with Mallory’s trichrome and examined using the Zeiss Axioskop 20 microscope. Detection of apoptosis was done using rabbit polyclonal antibodies to active caspase-3 (diluted 1:250, Abcam ab13847) in the paraffin sections from 3 diploid and 1 triploid males, mounted on Superfrost Plus microscope slides (Thermo Scientific). After deparaffinization and hydration of tissue sections, heat induced antigen retrieval was performed in 0.01 M citrate buffer, pH 6.0, at 97°C for 20 min. Afterwards tissues were washed in ×1 PBS (pH 7.4, 14 mM NaCl, 0.27 mM KCl, 1 mM Na2HPO4, 0.18 mM KH2PO4) with 0.05% Tween 20 (Sigma) (PBST), blocked in 6% BSA in PBST, and incubated overnight at 4°C with primary antibodies diluted in 3% BSA in PBST. Goat anti-rabbit secondary antibodies conjugated with ATTO 488 (SIGMA/MERCK) together with 1 μg/ml propidium iodide for DNA staining were applied for 1 h at RT. Washes of primary and secondary antibodies were performed 3 times in PBST for 10 min. Sections were mounted in ProLong Gold antifade reagent (Invitrogen, Life Technologies) and examined using Olympus FV1000 confocal microscope.

### Chromosome preparations

After dissection, gonads were hypotonized in 0,075 KCl for 20 min (adults) or for 10 min (tadpoles), fixed in two changes of Carnoy fixative (ethanol:glacial acetic acid 3:1) and kept under −20°C until use. For tadpoles gonads, we applied chromosome squashes technique to prepare metaphase chromosomes [modified from (Zaleśna et al., 2011)]. Gonads were placed in a drop of 70% acetic acid mounted between slide and coverslip and pressed. After freezing at −80°C for 20 min, coverslips were quickly removed with a scalpel and air-dried. Chromosomal spreads of adult testes and intestines were prepared from cell suspensions obtained after homogenization of tissue fragments in several drops of cold 70% acetic acid. A drop of the suspension was spread evenly with the pipette along the slide placed on a sloping hot-plate (60°C) (Dedukh et al., 2022a). The quality of the chromosomal preparations was checked under the phase contrast microscope (Zeiss Axiostar plus). Slides were stored in the freezer at −20°C. Genome composition of each preparation was determined on 10–20 metaphase plates.

### DNA extraction and labeling

Total genomic DNA was extracted from skeletal muscle tissue of four *P. lessonae* and four *P. ridibundus* males (Supplementary Table S1) using a standard phenol-chloroform protocol (Blin and Stafford, 1976). Whole genomes of both parental species were labeled either directly or indirectly using Nick Translation Kit (Roche Diagnostics) according to the protocol of Jenkins and Hasterok (Jenkins and Hasterok, 2007) with slight modifications. Direct labeling included ChromaTide Alexa Fluor 488-5-dUTP (Invitrogen, Life Technologies) and ChromaTide Alexa Fluor 546-14-dUTP (Invitrogen, Life Technologies). Indirect labeling used biotin-11-dUTP (Thermo Scientific) and digoxigenin-11-dUTP (Roche Diagnostics). The final volume of a reaction mixture (20 µl) contained 1 µg of genomic DNA of one of the parental species. Nick translations were carried out for 2.5 h to obtain the fragments about 100-200 bp in length. Reaction was checked by gel electrophoresis and stopped by the addition of 1 µl of 0.5 M EDTA (pH 8.0). Total genomic DNA from both parental species was also used to prepare unlabeled blocking DNA (100-500bp) by autoclaving in NewClave Autohouse AD7 for 5 min in 121°C.

Except for whole-genome probes, we also used FISH with centromeric satellite RrS1 probe, which enables clear distinction between *P. ridibundus* and *P. lessonae* chromosomes (Ragghianti et al., 1995; Ragghianti et al., 2004; Marracci et al., 2011). Probe was labeled with biotin-11-dUTP or digoxigenin-11-dUTP by PCR amplification in a C1000 Thermal Cycler (Bio-Rad, United States) according to Dedukh et al. (Dedukh et al., 2019; Dedukh et al., 2022b).

### *In situ* hybridization techniques

We applied genome *in situ* hybridization (GISH) and comparative genomic hybridization (CGH) combined with FISH (fluorescent *in situ* hybridization) with the use of RrS1 probe to identify the parental species genomes in germ line cells. FISH with telomeric probe was used to identify genome composition of tadpoles. During GISH, for each slide we mixed 300–500 ng of directly or indirectly labeled probe of one genome, unlabeled blocking DNA of another parental genome (15–20 times the excess of a labeled probe) and a single stranded blocking salmon sperm (Sigma-Aldrich), 30 times the excess of a labeled probe. During CGH [modified from (Doležálková et al., 2016)], for each slide we mixed differently tagged whole genome probes of both species in 1:1 ratio (300–500 ng per probe). Additionally, we used 300–500 ng of the labeled RrS1 probe to the mixture.

Prior the hybridization procedure, slides were pre-treated according to Schwarzacher and Heslop-Harrison (Schwarzacher and Heslop-Harrison, 2000) with 100 μg/ml DNase-free RNase A (EURx) and 1 μg/ml pepsin (Sigma-Aldrich) in 10 mM HCl for 8 min at 37°C in a humid chamber. Afterwards, slides were washed twice in ×2 SSC for 5 min and postfixed in 2% PFA in ×1 PBS for 10 min. After washing in ×2 SSC for 5 min, slides were dehydrated in ethanol series and air-dried.

Irrespective of the method of hybridization, DNA mixture was precipitated in 100% ethanol for 1 h at −80°C. Dried DNA mixture was dissolved in 15 µl of 100% deionized formamide (EuroClone) at 37°C for at least 1 h. For each slide, the hybridization mixture consisted of 50% deionized formamide, 10% dextran sulfate, ×2 SSC, 1% SDS, deionized water and resuspended DNA components. The final concentration of each probe was around 10–16 ng/μl dissolved in 30 µl used per slide. In case of FISH with telomeric probe, hybridization mixture included 40% deionized formamide, 12% dextran sulfate, ×2 SSC, 5 ng/μl single stranded (TTAGGG)_5_ probe conjugated with biotin and 10 to 50-fold excess of tRNA. Hybridization mixtures were pre-denatured for 6 min at 83°C (Bi and Bogart, 2006) and immediately chilled on ice for at least 10 min. Pre-denaturation step was omitted in FISH with telomeric probe. Hybridization mixture was put on slides, covered with coverslips and sealed with Rubbercement. Slides were denatured for 6 min at 74°C in Eppendorf ThermoStat Plus. Hybridizations were carried out for about 40 h (overnight in case of telomeric probe) in a dark humid chamber at 37°C (RT in case of telomeric probe).

Unless stated otherwise, all posthybridization washes were performed at 42°C. After hybridization, coverslips were carefully rinsed away with ×2 SSC. For FISH with telomeric probe we used 3 washes in ×2 SSC for 5 min at 44°C with shaking. Chromosomal slides after CGH, GISH and FISH with RrS1 probe were immersed twice in 15%–20% deionized formamide in ×0.1 SSC for 5 min each with a gentle shaking. Slides were additionally washed twice in ×2 SSC for 5 min each and 3 times in ×2 SSC for 3 min each. Directly labeled probes were washed in deionized water, dehydrated in an ethanol series, air-dried and counterstained with ProLong Gold antifade mounting reagent with DAPI (Invitrogen, Life Technologies). When using indirectly labeled probes or the mixture of directly and indirectly labeled probes, slides after dehydration step were washed once in ×4 SSC containing 0.2% Tween 20 for 5 min at RT and incubated in blocking solution including 5% BSA (BioShop) in ×4 SSC with 0.2% Tween 20 for 30 min at 37°C. Digoxigenin and biotin were detected by anti-digoxigenin-fluorescein (dilution 1:35 in blocking solution, Roche) and Streptavidin-Cy5 (dilution 1:35 in blocking solution, Invitrogen, Life Technologies) correspondingly. After incubation for 90 min at 37°C in a dark humid chamber, slides were washed three times in ×4 SSC with 0.2% Tween 20 for 8 min each, rinsed in deionized water for 3 min at RT, counterstained with 0.5 mg/ml DAPI (diluted 1:1000 in deionized water) for 1 h at RT in a dark chamber and finally mounted in ProLong Gold antifade reagent (Invitrogen, Life Technologies).

### Image processing

Chromosomal slides were analyzed under Olympus FV1000 confocal microscope equipped with FV10-ASW 2.0 software. Contrast and brightness adjustments were performed in FV10-ASW Viewer 4.2a or FIJI (Schindelin et al., 2012). Histological images were processed with CorelDraw Gaphics Suite 2017.

### Algorithm used for genotype assessment in the germ line cells

Metaphase plates were analyzed manually, number of *P. ridibundus* (R) and *P. lessonae* (L) chromosomes was counted based on whole genomic probes hybridization patterns and the number of RrS1 centromeric probe signals which resulted in bright fluorescence signals in 6 (5 big and 1 small) R chromosomes (Figure 6A, Supplementary Table S5). The assessment of genome composition of interphase nuclei based on the presence or absence of RrS1 signals on 6 R chromosomes. However, the elimination of L cannot be directly determined based on the use of the RrS1 probe. Whole-genome probes did not differentiate R and L chromosomes in dispersed interphase chromatin (Figures 6B,E). Although the analysis of interphase nuclei did not provide as precise and direct information on the composition of genomes, as in metaphase plates, it supplemented information on the elimination and endoreplication of R chromosomes and the genome type contained in micronuclei.

We divided cells into classes according to the number and composition of chromosomes: 1) with complete *n* number as haploid, diploid, triploid, tetraploid, 2) with incomplete number of chromosomes (*n* -) and assigned as aneuploids, 3) containing only one type of the genome, namely R or L, 4) or bearing chromosomes of two different genomes, they were defined as “mixed L+R”. Chromosomal plates with 12–13 chromosomes were estimated as haploid; chromosomal plates with 24–26 chromosomes were estimated as diploid; and consequently chromosomal plates with 36–39 chromosomes were estimated as triploid. The genome composition and ploidy of interphase cells was assessed according to the presence of RrS1 signals, namely, haploid R set had 5–6 signals. Each metaphase plate or interphase nucleus having the lower number of chromosomes in metaphase plates or RrS1 probe signals during interphase were estimated as aneuploids and were denoted by adding the prefix “hypo-” (hypo-haploid, hypo-diploid etc).

### Measurements of gametogenic cell sizes

We measured gonocytes in 12 tadpoles (4 RL, 4 RLL, 4 RRL), SSCs in 14 adult males (7 RL, 5 RLL, 2 RRL) and primary spermatocytes in 17 adult males (2 RR, 8 RL, 5 RLL, 2 RRL). The cell sizes were calculated from interactively drawn outlines (profiles) of the entire cells along the cell membranes (in case of G and SSC) or nuclear envelopes (in case of primary spermatocytes) using the computer program AxioVision (Zeiss). The size was defined as the equivalent circle diameter (d_c_) calculated from the measured area (A) of a cell or a nucleus [d_c_ = 2 ∙ √ (A/Π)] (Weibel, 1979).

For each gonad, we measured 100 undamaged cells for each gametogenic stage (G, SSC, spermatocytes and spermatozoa). In early larval gonads, where the total number of gonocytes was lower than 100, we measured all suitable cells from the whole series of paraffin sections. In adults, we measured SSCs and spermatocytes in a selected section. The size of spermatozoa heads was analyzed on chromosomal slides after GISH/FISH hybridization based on length and total area measurements performed in FIJI software (Schindelin et al., 2012).

### Statistical analysis

All statistical analyzes and some of the graphs showing the data from the measurements were using the CSS Statistica version 13 (StatSoft, Poland). The following tests were applied: Mann-Whitney U-test to compare two variables with unrelated features that do not have a normal distribution, and the ANOVA Kruskal-Wallis test to compare one feature among several datasets, supplemented with the post-hoc test. Descriptive statistics of measurements are given in Supplementary Table S13.

## Results

We compared the structure of testes as well as morphology and size of germ cells during ontogeny of gonads in tadpoles and in fully grown testes in adult males. First, we compared the differentiation stages between gonads of diploid and triploid hybrids. Second, we examined differences between gonocytes in tadpoles and SSCs in adults, focusing on the presence or absence of micronuclei which contain eliminated chromosomes (Chmielewska et al., 2018; Dedukh et al., 2020b). Third, we analyzed abnormalities of gonads including degeneration and death of germ line cells which may influence gonadal development. Finally, we assessed diploid and triploid hybrid male contribution into viable offspring by identification of progeny karyotypes.

### Morphology of testes and germ line cells

#### Histology of developing testes in tadpoles

**FIGURE 1:**
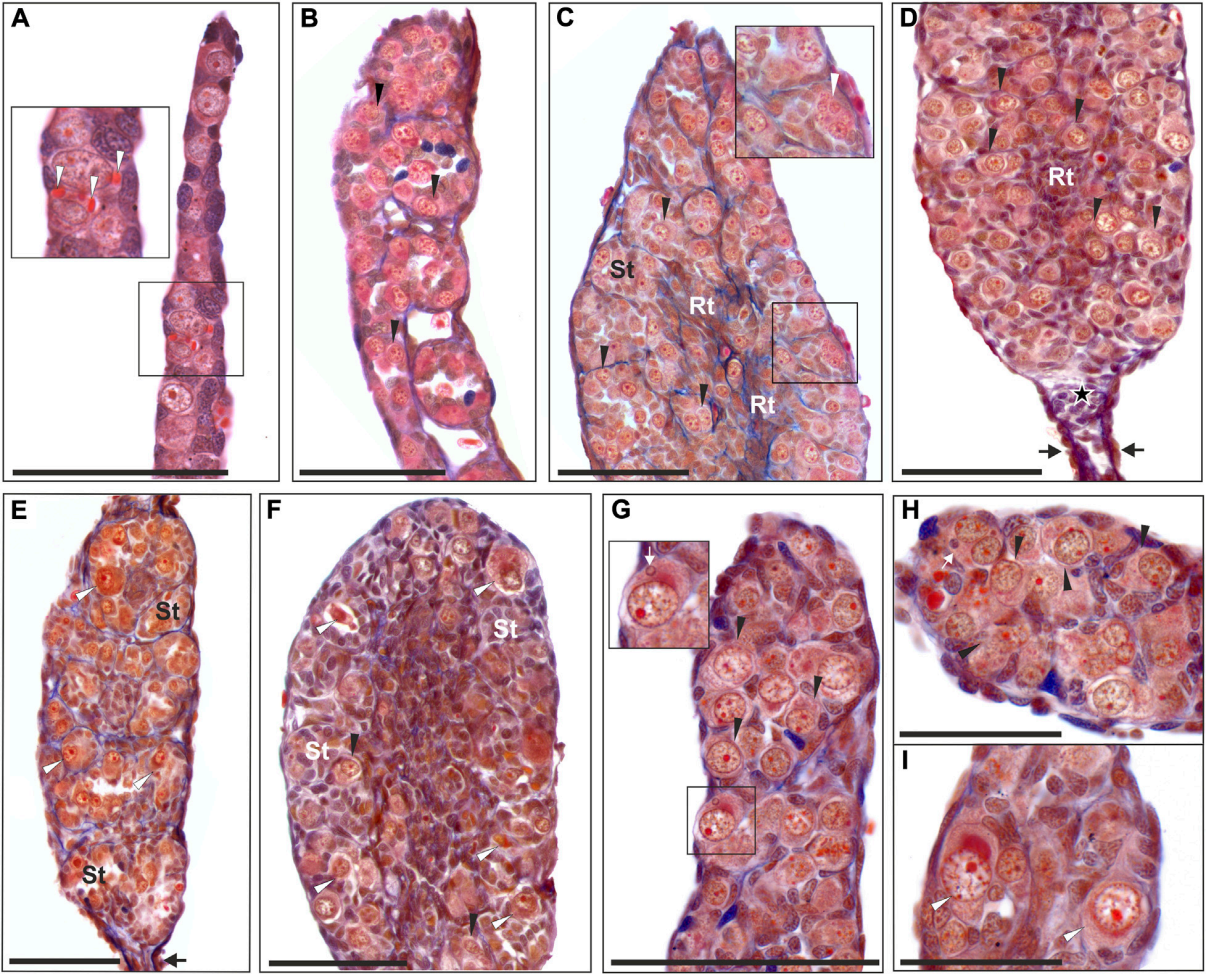
Morphology of tadpole gonads. Paraffin tissue sections of gonads stained with Mallory’s trichrome. **(A)** Undifferentiated gonad from RRL tadpole at 26 Gosner stage (G. st.) (# 207) with PGCs containing yolk platelets (white arrowheads). **(B)** Differentiating testis in RRL male at 31 G. st. (# 584) where gonocytes are invading the gonadal medulla and metamery is well preserved. **(C)** Young testis of RL individual at 38 G. st. (# 418) with regular seminiferous tubules (St) and rete testis (Rt) forming, containing numerous gonocytes, white arrowhead shows the abnormal cell with multinucleation (enlarged in the inset). **(D)** Young testis of RLL individual (G. st. 40, # 1352) with distal part preserved (black arrows) and a group of somatic cells left after degeneration of distal gonomers (asterisk), seminiferous tubules contain numerous gonocytes. **(E)** Testis of RL individual (G. st. 38, # 1499) with well-preserved metamery of the first 4 gonomers, seminiferous tubules (St) almost devoid of normal gonocytes, but with degenerating germ line cells (white arrowheads), black arrow points to distal part. **(F)** Almost sterile testis of the RLL male (G. st. 44, # 1170) during metamorphosis, filled with somatic cells, scarce normal germ line cells (black arrowheads) and abundant degenerating cells (white arrowheads) are present in seminiferous tubules; **(G)** Beginning from 29 G. st. (# 780) micronuclei can be detected in the cytoplasm of the gonocytes (white arrow), inset shows enlargement of the micronucleated cell, black arrowheads—gonocytes without micronuclei. **(H)** Some gonocytes have doubled cell nuclei and micronuclei (white arrow) (# 780). **(I)** Degenerating germ line cells (white arrowheads) are visible in gonads from early developmental stages (29 G. st.). Black arrowheads, normal gonocytes; St, seminiferus tubules; Rt, rete testis. Scale bar 100 µm.

We analyzed testes of 11 diploid (36–79 days after fertilization) and 16 triploid hybrid tadpoles with both RLL (10 individuals) and RRL (6 individuals) genotypes (18–84 days after fertilization) at Gosner stages 25–44, i.e. from beginning to feed until the final stages of metamorphosis (Supplementary Table S3). We found no differences in the testes development between diploid and triploid tadpoles, thereby we described them together. At stage 25, the gonads were sexually undifferentiated and contained Gs and sometimes few PGCs still containing yolk (Figure 1A). PGCs and Gs were localized in the cortical part around the somatic core of a gonad. Gonads were sexually differentiated beyond stages 28–30, when Gs in the testes migrated from the cortex to the gonad core (*medulla*) (Figure 1B). Concomitantly, future sperm collecting tubules (*rete testis*) were formed from the somatic medullar cells (Figures 1C,D). The proximal part of the early testis soon transformed into an functional oval gonad while the distal part degenerated (Figure 1D) corresponding to normal testis development (Haczkiewicz and Ogielska, 2013). After stage 40, mitotically dividing Gs were located in sex cords surrounded by a wall of medullar cells (Figure 1D), thus forming precursors of the seminiferous tubules. In 5 diploid and 3 triploid individuals, testes were underdeveloped in comparison to the rest of individuals and contained many degenerating Gs (Figures 1E,F, Supplementary Table S3). All Gs in the developing testes were morphologically similar (Figures 1B–H); they were spherical or ovoid, and the mean size of the whole cell was the smallest in diploid RL (16.39 µm), slightly larger in RRL (17.44 µm) and the largest in RLL (18.29 µm). The differences were statistically significant (Kruskal-Wallis test: H = 74.880, *p* < 0.001) between each of two groups compared among RL, RLL and RRL. The interphase nuclei occupied a central position and usually had 1–2 nucleoli (sometimes more) (Figure 1B). We observed single dividing Gs throughout the gonads and their number increased after stage 35. Per whole gonad we found 2–37 mitoses in diploids and 4–10 mitoses in triploids. In the cytoplasm of interphase Gs we found micronuclei (Figures 1G,H), and the number of micronucleated cells per gonad varied between 1 and 65 in diploids and 1–20 in triploids.

#### Histology of testes in adults

**FIGURE 2:**
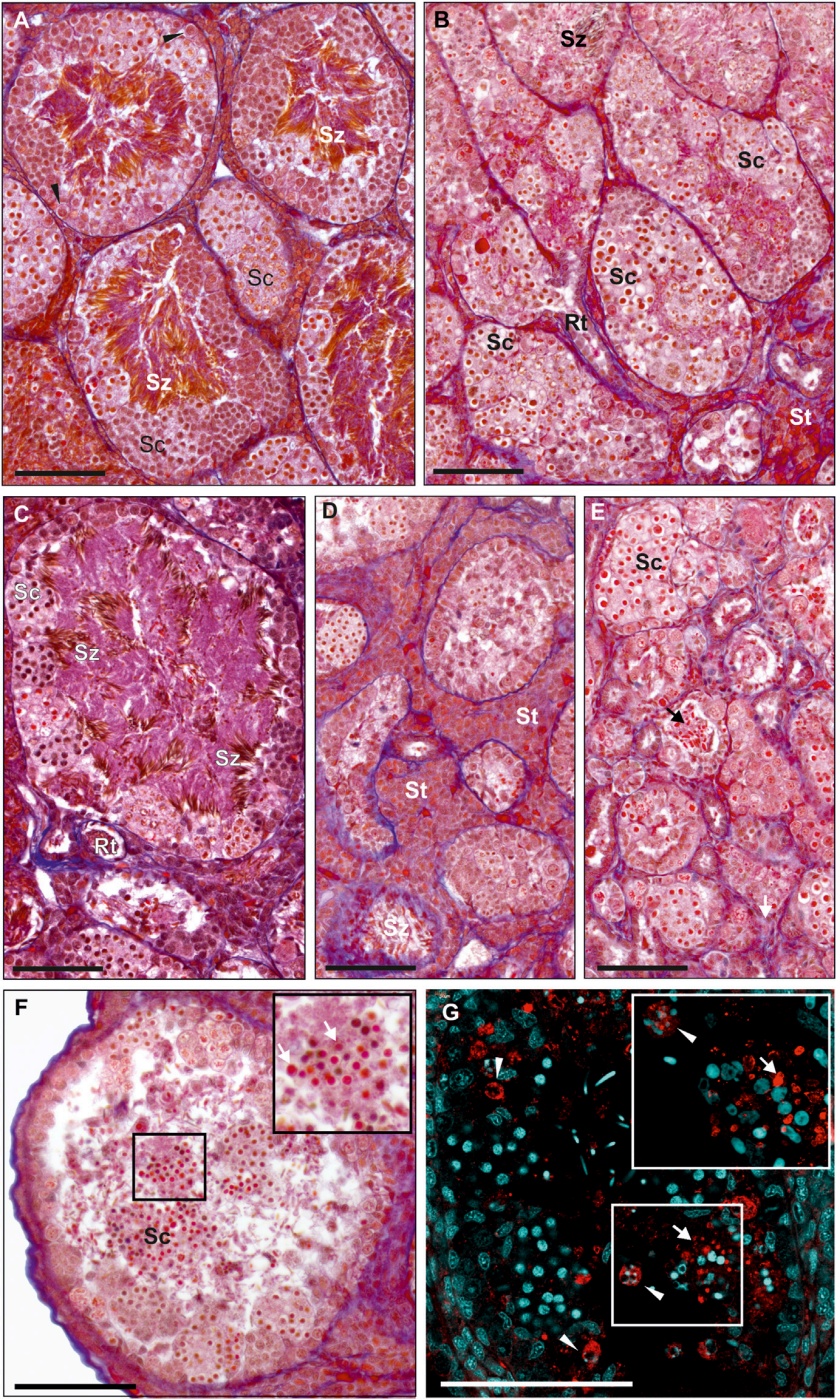
Morphology of testes from adult males. Paraffin tissue sections of gonads stained with Mallory’s trichrome. **(A)** Normal testis in the *P. ridibundus* individual (# 42/15), where seminiferous tubules are filled with spermatozoa (Sz) and many spermatocytes (Sc), as well as regular SSCs (black arrowheads). **(B)** Normal testis in the RL male # 7, tubules are filled with spermatocytes (Sc) varying in size, low amounts of spermatozoa are visible (Sz), properly developed *rete testis* (Rt) and somatic tissue (St). **(C)** Normal testis in the RRL male # 13, cysts filled with spermatocytes (Sc) at the tubule wall and numerous spermatozoa visible in the tubular lumen (Sz). **(D)** Abnormal testis of the RL individual # 2 with hypertrophy of interstitial tissue (St, Somatic tissue), seminiferous tubules contain low amount of spermatogenic cells. **(E)** Abnormal testis of the RL male # 9, seminiferous tubules have abnormally low tubule diameter, some tubules have no germ line cells, very few spermatozoa can be found but they have abnormal shape (black arrow). **(A–E)** Images shown at the same magnification to ease comparison of the seminiferous tubules dimensions. **(F** and **G)** Degeneration of whole spermatogenic cysts filled with spermatocytes (Sc), detached from tubule wall and apparently present in the tubule lumen, RL male # 1. **(F)** Histological image, inset at the top right corner of the image shows enlargement of the region framed in the center of the image, degenerating cells (white arrows). **(G)** Immunohistochemistry with active caspase-3 (red), DNA (cyan), inset at the top right corner shows higher magnification image of the same region framed with white box in the center of the image, activity of caspase-3 can be detected both in the degenerating spermatocytes in cysts (white arrow) and in SSC (white arrowheads) containing apoptotic bodies filled with chromatin. Scale bar 100 µm.

Typically, testes of the parental species, here *P. ridibundus*, were composed of seminiferous tubules separated by interstitial tissue and *rete testis* (Figure 2A). Hybrid testes, both diploid and triploid, were usually impaired (Figures 2D,E). Seminiferous tubules varied along their length and their particular portions differed in diameter and germ cell number and type, thus on a cross section of a testis we could observe both regular (Figures 2B,C) and abnormally narrow, usually sterile (Figure 2E) or enlarged tubules (males # 1, 13, 14). Normal tubules were surrounded by a thin layer of interstitial tissue, while interstitial tissue around sterile portions was thick and massive (Figure 2D) (males # 3, 8, 14).

**FIGURE 3:**
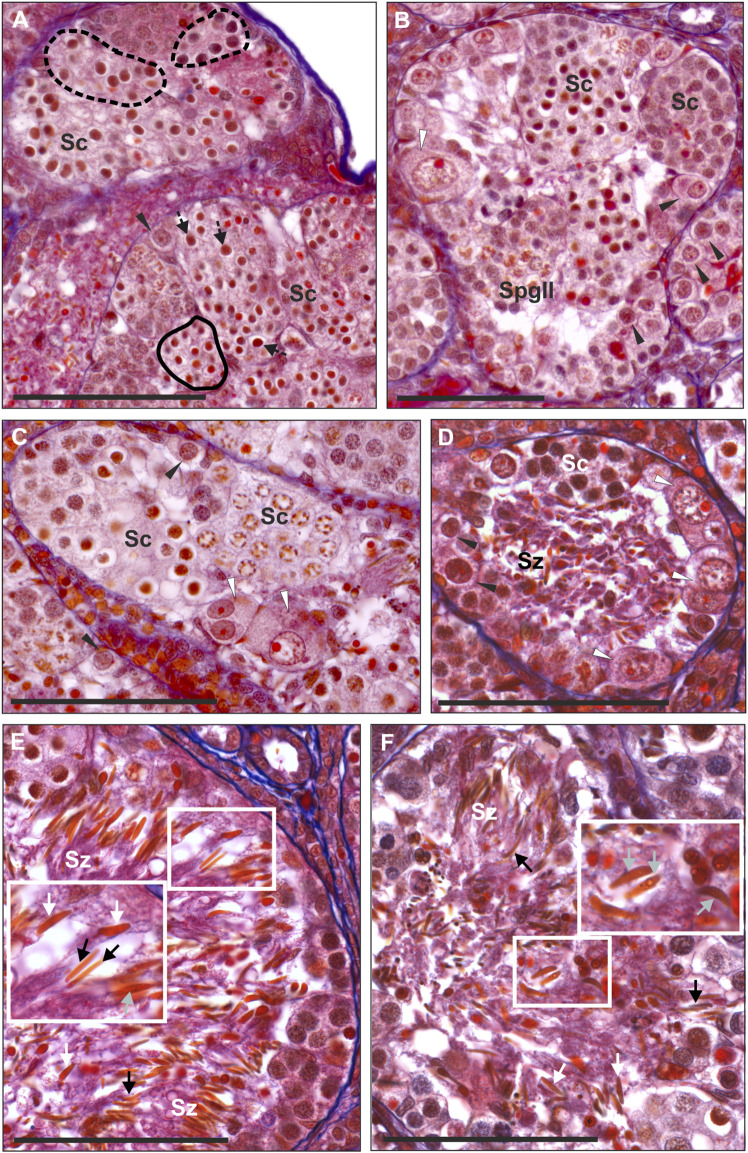
Morphology of spermatogenic cells in testes from adult males. Paraffin tissue sections of gonads stained with Mallory’s trichrome. **(A)** Spermatocytes (Sc) in seminiferous tubules have different sizes, large spermatocytes can be found in separate cysts (outlined by a dashed line) or can be scattered singly (black arrows with a dashed line) among regular spermatocytes, normal size spermatocytes forming uniform cysts (outlined by a solid line), normal SSC (black arrowheads) in the testis of the RL male # 1. **(B)** Seminiferous tubule of the RLL male # 12 with spermatogenic cysts filled with spermatocytes (Sc) at the leptoten stage (at the right) or at zygoten stage (in the center) of meiotic prophase I, cyst of secondary spermatogonia (SpgII), normal SSCs (black arrowheads) and abnormal SSC (white arrowhead) at the tubule wall. Abnormal SSC is swollen, chromatin is irregular and nucleolus is enlarged, probably as a sign of necrosis. **(C)** Fragment of the seminiferous tubule from the testis of RL male # 5 (fertile father RL from the crossing experiment) filled with spermatocytes (Sc) at leptoten/zygoten stage (seen on the left side) or late pachyten (seen on the right side), binucleated and abnormally large necrotic SSCs at the tubule wall (white arrowheads). **(D)** Seminiferous tubule of the RL male # 8 exhibiting many abnormal and degenerating binucleated SSCs (white arrowheads) and degenerating spermatozoa (Sz) in the tubule lumen. **(E** and **F)** Some males produce spermatozoa varying in size. **(E)** RRL male # 18 (fertile father RRL from the crossing experiment) has large (white arrows) and normal size (black arrows) spermatozoa in the seminiferous tubule. **(F)** RL male # 6 (fertile father RL from the crossing experiment), normal size spermatozoa (black arrows), some large spermatozoa have signs of degeneration (grey arrows). Insets in **E** and **F** show enlarged views of the framed area with large spermatozoa. Black arrowheads—normal SSCs. Scale bar 100 µm.

We found a whole spectrum of spermatogenic cells, i.e., SSCs, secondary spermatogonia, meiotic spermatocytes, spermatids and spermatozoa (Figures 3A–F). As spermatogenesis in water frogs is asynchronous, we could follow all stages of the process, although the proportion of SSCs, cysts and spermatozoa depended on time point of the reproduction cycle at which the tissue was fixed. We observed the abundance of mitotically dividing SSCs (Supplementary Figure S2A). Mitotic cells showed the lack of spindle tubules after administration of colchicine to the males before preparation of the gonads for histology and GISH study. In diploid and triploid males, we observed that SSCs were situated singly against the tubule wall. Each SSC was wrapped by accompanying Sertoli cells that later would form a cyst wall, inside which meiosis proceeded. After several mitotic divisions, a single SSC gave rise to a cluster of secondary spermatogonia (Figure 3B) that transformed into primary spermatocytes. We observed all stages of spermatocytes, from leptotene/zygotene, pachytene, diplotene (Figures 3A–C), secondary spermatocytes, and then spermatids and spermatozoa (Figures 3D–F) that were released into the seminiferous tubule lumen after the cysts opening during spermiation, initially in bundles, then dispersed (Figures 3E,F).

Normal SSCs had single ovoid or spherical nuclei with distinct nucleoli (Figures 3A,B). Neither of SSCs contained micronuclei and this feature clearly distinguished them from G cells in tadpoles. Mean diameter of SSCs in diploids was 14.79 µm, in triploid RLL—15.38 µm and RRL—15.90 µm, and values differed statistically (Kruskal-Wallis test: H = 46.555 *p* < 0.001) between each of two groups compared among RL, RLL and RRL.

**FIGURE 4:**
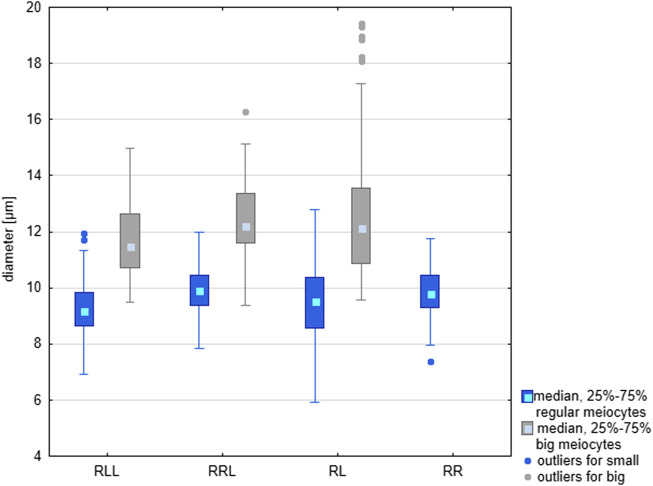
Comparison of the size of regular and large meiocytes in the parental species (RR), diploid (RL) and triploid RLL and RRL hybrids. Differences in size are statistically significant according to the results of Mann-Whitney U-test. RL: Z = −19.821, *p* < 0.001: RLL: Z-16.011, *p* < 0.001; RRL Z = −12.618, *p* < 0.001.

Cysts with secondary spermatogonia (Figure 3B) were situated close to SSCs and were not numerous due to short mitotic cycles, as opposed to cysts containing meiotic cells, especially long-lasting pachytene spermatocytes (Figures 3A–C). We noticed that spermatocytes varied in size, some of them being clearly bigger. We found whole cysts filled with only big spermatocytes or cysts with regular spermatocytes with admixture of big ones (Figure 3A); such cysts were distributed throughout all tubules singly or in groups (Figure 3A). The mean size of nuclei of regular spermatocytes at the bouquet stage differed significantly among various genotypes (Kruskal-Wallis test: H = 94.135, *p* < 0.001), with one exclusion, a difference between RR and RRL was not statistically significant. Nuclei were the smallest in RLL—9.26 µm, slightly bigger in RL—9.40 and RR—9.85, and the biggest in RRL—9.88 µm. The mean diameters of nuclei in big spermatocytes were 12.49 µm in RL, 11.69 µm in RLL and 12.44 µm in RRL (Figure 4). We found the statistically significant difference of the diameters between normal and big spermatocytes within each group of RL (Mann-Whitney U-test, Z = −19.821, *p* < 0.001), RLL and RRL males (in RLL Z-16.011, *p* < 0.001, in RRL Z = −12.618, *p* < 0.001). The frequency of big spermatocytes in diploids ranged from 16.08 % to 36.99% (mean 25.77%), whereas in triploids from 8.93% to 45.93% (mean 19.88%).

Spermatozoa were situated inside the tubule lumen. We noticed that males with regular and big SSCs and spermatocytes had also regular and big spermatozoa (Figures 3E,F). In 6 diploids (## 2, 3, 4, 9, 10, 11) spermatozoa were very scarce, in 3 (## 1, 5, 10) were uniform and regular in size and in 3 (## 6, 7, 8) had admixture of clearly big ones (Figure 3F). Among triploids, 1 RLL (# 12) (Figure 3E) and 2 RRL (## 17, 18) had regular and big spermatozoa, 3 RLL (## 13, 14, 15) had mostly regular ones, only in male # 16 we noticed neither big meiocytes, nor big spermatozoa although we observed some clearly bigger SSCs.

Sperm size, expressed by the area of their head surfaces (measured on the same spread preparations which were used in GISH analysis—see below), differed between diploids and two kinds of triploids (Kruskal-Wallis Test: H = 41.067, *p* < 0.001). Spermatozoa in RRL had the biggest mean area (29.6 µm^2^), moderate in RLL (26.33 µm^2^), and the smallest in RL (17.9 µm^2^). The largest variation in size was observed in triploids (ranges 8–98 μm^2^ in RLL and 7–89 μm^2^ in RRL) and the smallest in diploids (9–40 μm^2^). Big spermatozoa were assessed as diploid, as was clearly seen in spermatozoa with two RR chromosome sets (12 RrS1 signals) in comparison to regular ones with only one R set according to FISH-based genome identification (Supplementary Figure S3). Such a variety of sizes of spermatogenic cells was never observed in the parental species.

#### Abnormalities and degeneration

Besides normally looking germ line cells we detected various numbers of abnormal ones, both in tadpoles and in adults of all ploidy levels. The degenerating Gs and SSCs were similar in morphology: they had shrunken cytoplasmic content, larger size than normal cells, degenerated chromatin with irregular distribution and enlarged nucleoli (Figures 1E,F,I, 3B–D). They were often bi- or multinucleated with 2–7 spherical nuclei per cell (Figures 1C, 3C,D), among which 1 or 2 nuclei were larger than others (4–8 µm in diameter) (Figure 1H).

**FIGURE 5:**
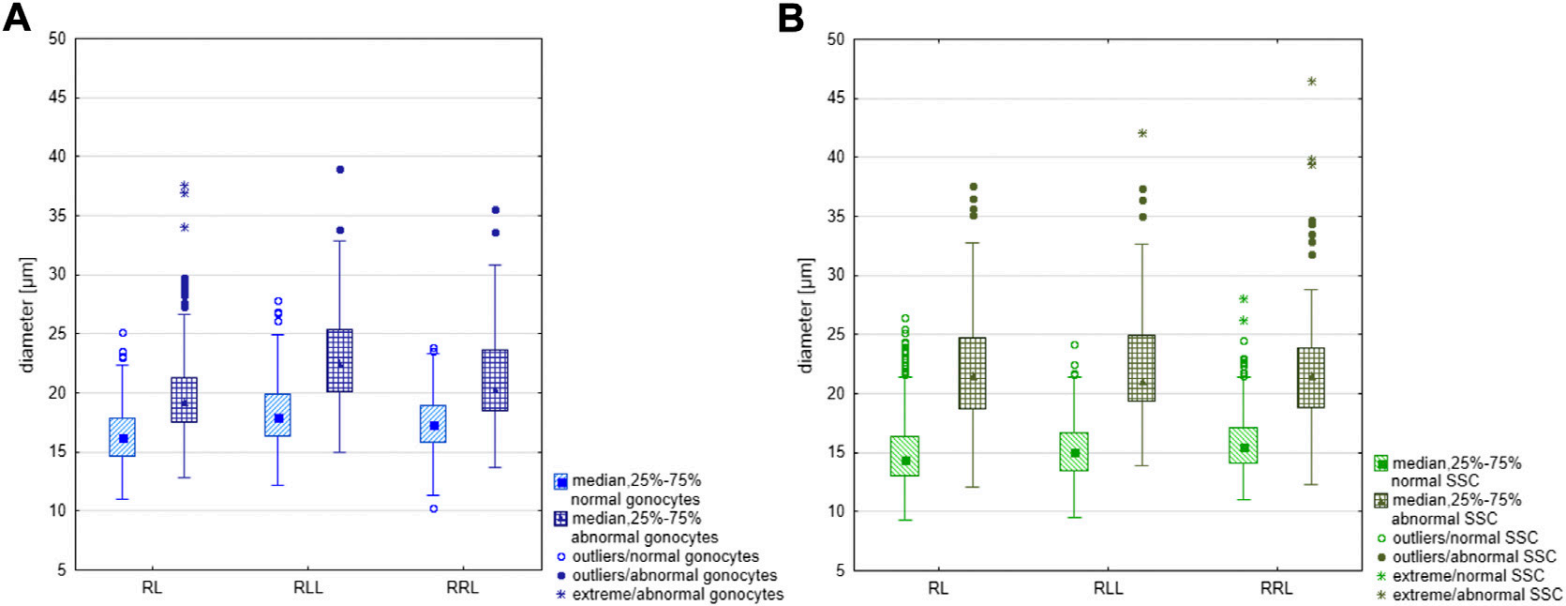
Comparison of the size of regular and large gonocytes and SSCs in diploid (RL) and triploid RLL and RRL hybrids. **(A)** Difference in size between regular and large gonocytes was statistically significant according to the Mann-Whitney U-test, RL: Z = −11.464: RLL: Z = −7.502 and RRL Z = −9.455, *p* < 0.001. **(B)** Difference in size between regular and large SSCs was statistically significant according to the Mann-Whitney U-test, RL: Z = −18.119, *p* < 0.001; RLL: Z = −13.121, *p* < 0.001; RRL: Z = −10.8660, *p* < 0.001.

In tadpoles, degenerative Gs (Figures 1E,F) appeared from stage 30 onwards, although we observed a single degenerated G in an individual as early as at stage 25 (476/30b/15). Typically, the intensity of degeneration was up to several dozen cells per gonad and the more degenerating cells, the more impaired was the gonad, i.e. small and underdeveloped (as described in the first section). Degenerating G cells were bigger than normal ones (means: RL—19.92 µm, RRL—21.05 µm and RLL—22.94 µm) (Figure 5A) and the differences were statistically significant (Mann-Whitney U-test, RL: Z = -11.464: RLL: Z = −7.502 and RRL Z = −9.455, *p* < 0.001). The frequency of abnormal Gs was higher in diploid individuals, and ranged from 31.8 to 47.3% (mean 39.55%), while it was lower in triploid individuals and ranged from 13.9% to 34.1% (mean 24%) in RLL and from 15.7 to 37.0% (mean 26.35%) in RRL. In tadpoles we observed numerous abnormal mitoses (Supplementary Table S3). Some of them had lagging chromosomes between the two masses of segregating chromosomes during anaphase or misaligned chromosomes outside the metaphase plate (Supplementary Figure S2B) or had multipolar karyokinetic spindles (Supplementary Figure S2C).

In adult males, degenerated single SSCs and whole cysts with degenerated meiocytes were often detached from seminiferous tubule wall and were lost in the lumen (Figures 2F,G). All sorts of spermatogenic cells in adults suffered from cell death, as confirmed by active caspase-3 assay (Figure 2G) in 3 diploid and 1 RRL males (## 1, 6, 8, 14). We observed accumulation of caspase-3 signal in cytoplasm of some SSCs with apoptotic bodies containing condensed chromatin, or in spermatogenic cysts where cells had condensed nuclei. Abnormal SSCs were bigger than regular ones (mean diameter: 22.15 µm in RL, 22.52 µm in RLL and 22.44 µm in RRL) (Figure 5B); the biggest ones were up to 46.51 µm. The difference in size between normal and abnormal SSCs were significant in each group of studied males (Mann-Whitney U-test: RL: Z = −18.119, *p* < 0.001; RLL: Z = −13.121, *p* < 0.001; RRL: Z = −10.8660, *p* < 0.001). The frequency of abnormal SSCs varied among individuals and ranged from 6.47% to 33% (mean 19.74%) in diploids and from 1.72% to 33.73% (mean 17.73%) in triploids.

The high frequency of degenerating SSCs and meiocytes resulted in the reduced number of spermatozoa, as was the case of 3 diploid (## 2, 4 and 9) and 1 triploid (# 14) adult males. Spermatozoa released into tubule lumen also displayed abnormalities. Their nuclei (sperm heads) had irregular shapes (round, oval or spindle-like) and contained heterogeneous chromatin with small dots (Figures 3E,F); the abnormal spermatozoa eventually degenerated (Figure 3E).

### Chromosome number and composition of gonocytes in diploid and triploid tadpoles

#### Diploid RL tadpoles

We analyzed 316 cells (106 metaphase chromosomal plates and 210 interphase nuclei) from 21 tadpoles at stages 25–45 (Supplementary Tables S4, S5).

**FIGURE 6:**
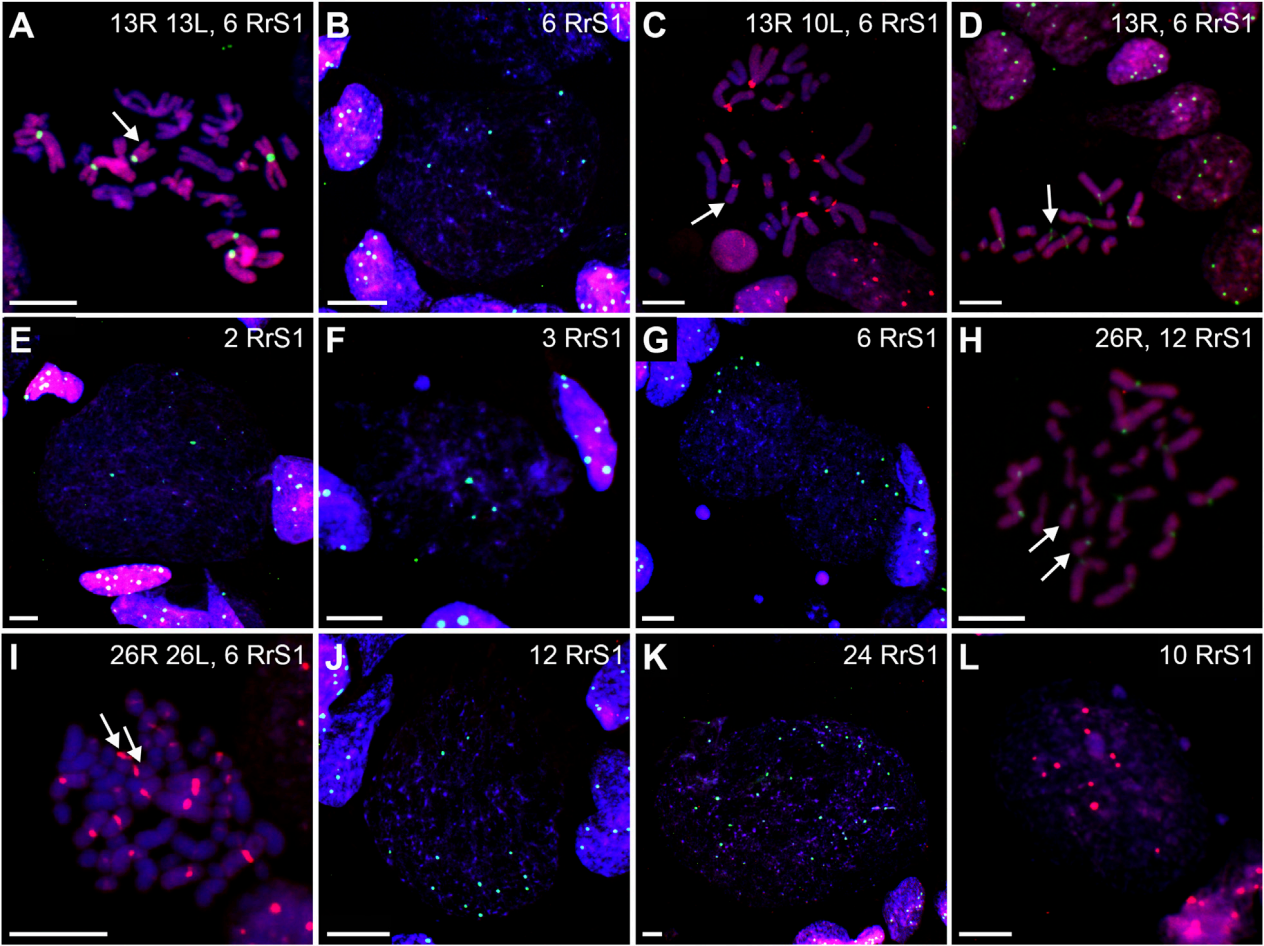
Genomic status of gonocytes in gonads of diploid RL tadpoles. Chromosomal preparations from gonadal squashes were probed with **(A,B, D–H, J,K)** whole genomic *P. ridibundus* probe Cy5 (red) and RrS1 *P. ridibundus* pericentromeric probe labelled with FITC (green) or **(C,I,L)** with whole genomic *P. ridibundus* and RrS1 probes labelled with Cy5, DNA stained with DAPI (blue). Somatic cells display 5-6 RrS1 signals **(B, D–J)**. Arrow - medium-small heterobranchial submetacentric chromosome (8) with centromeric RrS1 signal. **(A)** metaphase before elimination with 13R 13L chromosomes and 6 RrS1 signals, note 6 centromeric RrS1 signals and only 1 medium-small chromosome with centromeric signal (white arrow), G. st. 40, # 1518. **(B)** interphase before elimination, note 6 RrS1 signals, G. st. 36, # 1299. **(C)** Metaphase 13R 10L chromosomes during elimination of L genome, 1 large and 2 small L chromosomes are lacking, G. st. 31, # 307. **(D)** haploid set of 13 *P. ridibundus* chromosomes after elimination of L genome, G. st. 36, # 1299. **(E)** gonocyte eliminating R genome, note 3 RrS1 signals present instead of 6, G. st. 36, # 1423. **(F)** Elimination of R genome, note 3 RrS1 signals in main nucleus, and micronucleus without signal, probably eliminating R chrosomes without signal, G. st. 40, # 1518. **(G)** Primary nuclei displaying no elimination and 6 RrS1 signals, micronuclei without RrS1 signal, probably eliminating L chromosomes, G. st. 40, # 1518. **(H)** metaphase with 26 *P. ridibundus* chromosomes after elimination of L genome and endoreplication of R genome, G. st. 38, # 1525. **(I)** metaphase without elimination but with endoreplication of genome to 4n RRLL, note 12 strong RrS1 signals and 10 large *P. ridibundus* chromosomes, and 10 large *P. lessonae* chromosomes without the signal, G. st. 29, # 178. **(J)** large interphase nucleus, Ø 65µm, endoreplication of R genome to 2n, note 12 RrS1 signals, G. st. 36, # 1299. **(K)** giant interphase Ø 130 µm, endoreplication of R genome to 4n, note 24 RrS1 signals, G. st. 36, # 1299. **(L)** interphase during endoreplication with 10RrS1 signals and 3 micronuclei without signal, G. st. 40, # 1515. Scale bar 10 µm.

**FIGURE 7:**
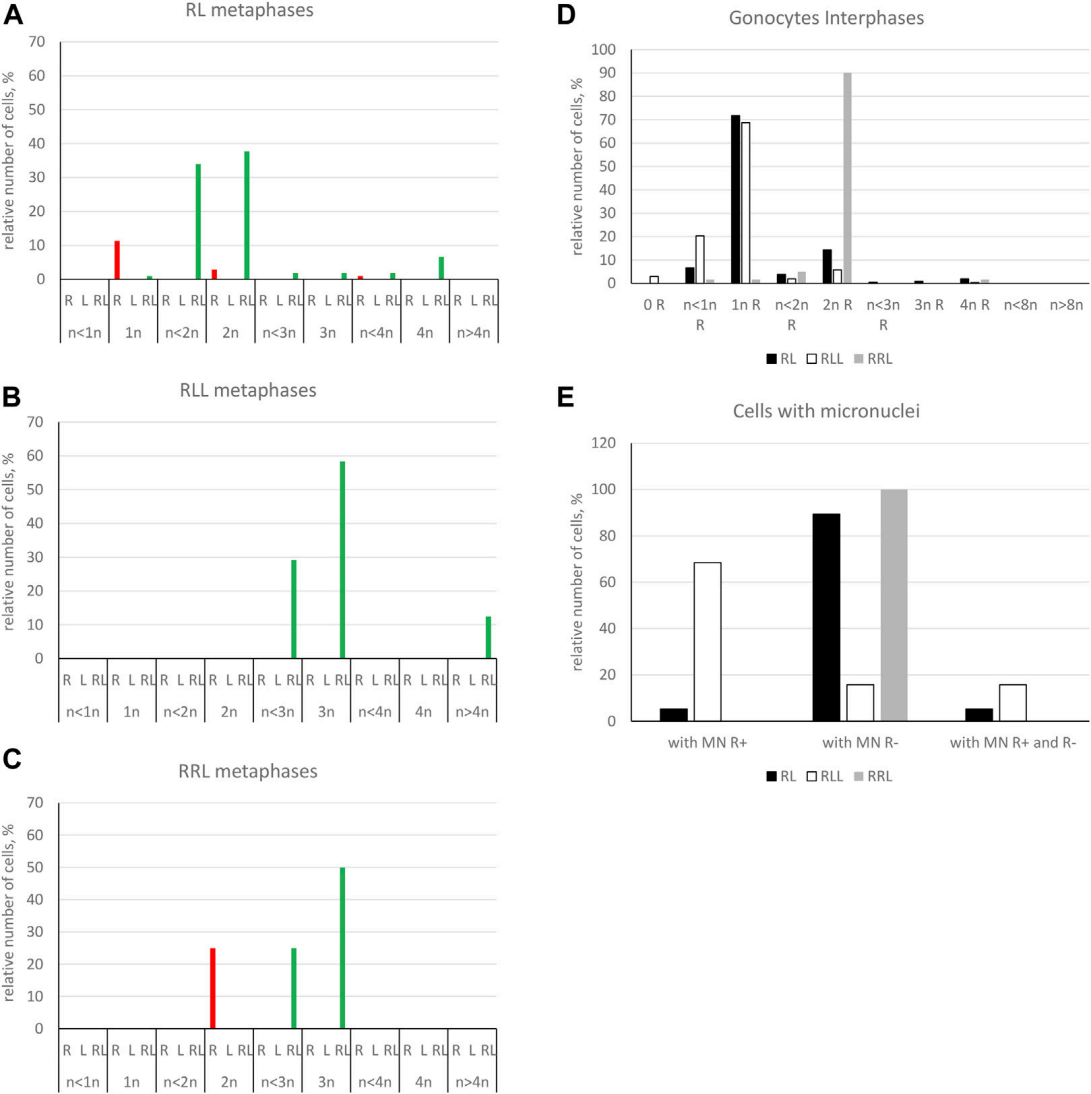
Genomic composition of gonocytes during phase of genome elimination in tadpoles. Relative number of cells displaying various genomic compositions according to GISH/FISH analysis of chromosomal spreads. **(A)** RL metaphase plates. **(B)** RLL metaphase plates. **(C)** RRL metaphase plates. **(D)** Interphase gonocytes in 2n and 3n tadpoles. **(E)** Cells with micronuclei possessing RrS1 signal or without signal in 2n and 3n tadpoles.

We found 58.51% of metaphase plates with regular number of chromosomes. Among those cells, 37.76% were diploid RL (assessed as cells before genome elimination) (Figures 6A, 7A), 11.33% were haploid R (assessed as after elimination of L and before or no endoreplication, Figure 6D), 2.82% were diploid RR (assessed as after elimination of L and endoreplication of R genome, Figure 6H), 6.60% were tetraploid RRLL (assessed as without genome elimination, but after endoreplication of both chromosomal sets, Figure 6I). We did not find diploid LL, suggesting the absence of R genome elimination in all gonocytes in our sample. The remaining gonocytes (41.49%) were aneuploid, of which the majority (33.97%) were hypo-diploid mixed R+L (Figure 6C) and the remaining 7.52% represented other classes of aneuploidy. Among hypo-diploid plates, we found metaphases with lower number of R chromosomes (N = 11 plates) (Supplementary Table S6), with lower number of L chromosomes (N = 12 plates, Figure 6C), or with depletion of both R and L chromosomes (N = 13). We suggest that these cells eliminate R or L genomes, or both genomes, respectively.

Furthermore, in 71.87% of interphase nuclei, we observed 5–6 RrS1 signals (Figure 6B, Figure 7D) (Supplementary Table S5). Despite we were unable to discriminate L genome, we suggest that these cells were diploid RL (assessed as cells before genome elimination). However, we cannot exclude that they were haploid R, as we found haploid cells during metaphase. Next, 14.29% cells displayed 10–12 RrS1 signals (Figure 6J), which may mean both RR cells (assessed as cells after elimination of L and endoreplication of R genome), and RRLL cells (assessed as cells without genome elimination and after genome endoreplication). A small fraction of gonocytes (1.92%) had 20–24 RrS1 signals that indicated two rounds of endoreplication of R genome (RRRR, Figure 6K). We did not find gonocytes with no RrS1 signals, i. e. after complete elimination of R. The remaining gonocytes (11.44%) were aneuploid, among which 6.67% were hypo-haploid R, probably during elimination of R genome (Figure 6E), 3.81% were hypo-diploid R, probably during endoreplication of R, and 0.96% were triploid RRR.

In 5 out of 21 tadpoles, we found 1–3 micronuclei in interphase cells (N = 19, 9.05% of all cells) (Supplementary Table S7), which is an evidence of genome elimination at this stage of gonad development. Only two micronuclei (7.41%) contained the eliminated R chromosomes (displayed RrS1 signals), whereas the most of them (N = 25, 92.59%) had no RrS1 signal (Figures 6F,G,L, 7E). This suggests that the majority of gonocytes eliminates L chromosomes and is consistent with the results obtained in metaphases; however, micronuclei without RrS1 signal may also contain small R chromosomes. Moreover, 12 of 19 gonocytes with micronuclei had 6 RrS1 signals in the main nuclei (Figure 6G), which indirectly confirmed the elimination of L genome. Only 5 cells had 2-5 RrS1 signals in the main nucleus, showing R genome elimination (Figure 6F). Interestingly, 2 cells had 7 and 10 RrS1 signals, witnessing ongoing R genome endoreplication, while they eliminated chromosomes without signal in 2-3 micronuclei (Figure 6L), which may suggest that genome elimination may occur also after genome endoreplication.

#### Triploid RLL tadpoles

We analyzed 334 gonocytes (24 metaphase chromosomal plates and 310 interphase nuclei) from 10 tadpoles at stages 32-40 (Supplementary Tables S4, S5).

**FIGURE 8:**
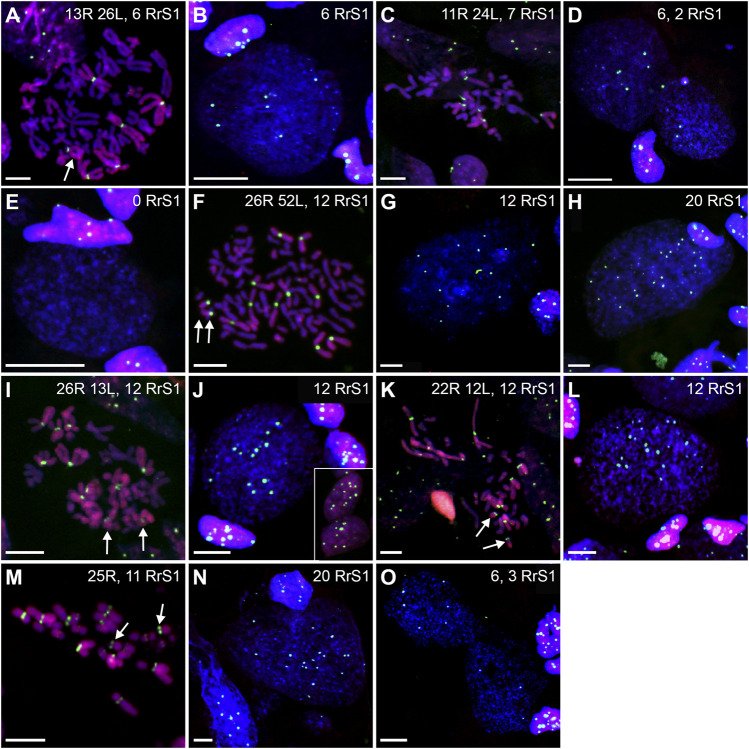
Genomic status of gonocytes in gonads of triploid RLL and RRL tadpoles. Chromosomal preparations from gonadal squashes from triploid RLL **(A–H)** and RRL **(I–O)** tadpoles were probed with **(A–O)** whole genomic *P. ridibundus* probe Cy5 (red) and RrS1 probe labelled with FITC (green). Somatic cells display 5-6 RrS1 signals in RLL **(B,D,E,H)** or 10-12 RrS1 signals in RRL **(J,L,N)**. Arrow - medium-small heterobranchial submetacentric chromosome (8) with centromeric RrS1 signal. **(A)** metaphase before elimination, arrow points to medium-small R chromosome with centromeric signal, G. st. 37, # 1115. **(B)** interphase before elimination, note 6 RrS1 signals, G. st. 37, # 1115. **(C)** metaphase with 11R and 24L chromosomes, possibly during elimination of R genome, 7 RrS1 signals, G. st. 38, #, 1226. **(D)** Gonocytes at different stages, one without elimination, other eliminating R genome, note the micronucleus eliminating R chromosome bearing RrS1 centromeric signal, G. st. 37, # 1115. **(E)** Interphase gonocyte without RrS1 signals, Ø 32 µm, G. st. 37, # 1115. **(F)** Endoreplication of RLL chromosome set to 6n, note 10 large chromosomes with RrS1 signals, and 10 large chromosomes without signal belonging to L genome, G. st. 26, # 273. **(G)** Cell after one round of endoreplication to 12 RrS1 signals, Ø 55 µm, G. st. 38, # 1226. **(H)** aneuploid interphase with 19 RrS1 signals witnessing incomplete two rounds of genome endoreplication, Ø 55 µm, G. st. 32, # 825. **(I)** RRL male, metaphase before elimination, 12 *P. ridibundus* chromosomes showing RrS1 signals, arrow points to small acrocentric R chromosomes, G. st. 37, # 1007. **(J)** interphase before elimination, inset shows somatic cells with 10-12 RrS1 signals, G. st. 41, # 1643. **(K)** mitosis with elimination of R genome, 22R and 12L chromosomes, G. st. 37, # 1007. **(L)** Interphase gonocyte with 12 strong RrS1 signals and micronucleus without RrS1 signal during elimination of L genome, G. st. 41, # 1643. **(M)** metaphase after elimination of L chromosomes, 25R chromosomes, 11 having RrS1 signals, one big R chromosome is lacking, G. st. 41, # 1643. **(N)** large interphase nucleus ø 60 µm with 20 RrS1 signals after endoreplication, G. st. 37, # 1007. **(O)** Abnormality - elimination of R genome, first cell with 3 and second cell with 6 RrS1 signals, note surrounding somatic cells nuclei with 10-12 RrS1 signals, G. st. 41, # 1643. Scale bar 10 µm.

We found 58.33% metaphase plates with regular number of RLL chromosomes (3*n* = 39 chromosomes), suggesting no elimination and no endoreplication (Figures 7B, 8A). The rest of metaphase cells (41.67%) were aneuploid, of which 29.17% were hypo-triploid (Figure 8C) and 12.50% hypo-hexaploid and hexaploid (23–26R and 43–50L chromosomes, Figure 8F). Hexaploid cells were probably the result of endoreplication without prior R genome elimination. We did not find any metaphases after complete genome elimination; however, hypo-triploid plates may represent cells during genome elimination (Supplementary Table S6).

Among interphase nuclei, the prevailing portion (68.71%) was assessed as RLL (5-6 RrS1 signals) (Figures 7D, 8B) suggesting that these cells are before elimination of R genome. Only 2.90% of gonocytes had no RrS1 signals (Figure 8E) and most probably were LL, i.e., after the elimination of R genome. We observed 5.81% of cells with 10–12 signals (Figure 8G) and 0.32% of cells with 20 signals (Figure 8H) suggesting one or two rounds of endoreplication, correspondingly. The remaining 22.26% of interphase nuclei were aneuploid, hypo-haploid R (Figures 7D, 8D), probably during R genome elimination, and hypo-diploid R, probably during R genome elimination after whole genome endoreplication.

In six individuals, we found 1–5 micronuclei in 19 interphase gonocytes (6.13%) (Supplementary Table S7). The vast majority of the micronuclei (70.59%, N = 24) had 1-2 RrS1 signals (Figures 7E, 8D) and the remaining 29.41% (N = 10) had not. The presence of RrS1 signals in micronuclei and the reduced number of these signals (1–4) in the main nuclei indicates that elimination of R genome in RLL tadpoles prevailed, which is consistent with the presence of hypo-haploid cells during interphase.

#### Triploid RRL tadpoles

We analyzed 73 gonocytes (12 metaphase chromosomal plates and 61 interphase nuclei) from 5 tadpoles at stages 34–41 (Supplementary Tables S4, S5).

Predominating portion of metaphase plates (75.00%) had regular chromosomal compositions: a half of them (N = 6, 50.00%) were RRL (3*n* = 39 chromosomes, Figures 8I, 7C), i.e., before elimination and endoreplication, and 25.00% (N = 3) had diploid number of R chromosomes (Figure 8M), which indicates the complete elimination of L genome. Aneuploid chromosomal compositions (25.00%) were hypo-triploid with lower number of R chromosomes (Figure 8K) and/or L chromosomes (Supplementary Table S6), which may suggest R or L genome elimination.

Most frequently we found interphase nuclei (90.16%) displaying 10-12 RrS1 signals (Figures 7D, 8J), which indicates no elimination of R chromosomes. In one nucleus (1.64%) we found 20 RrS1 signals (Figure 8N), which indicates endoreplication of R genome. Remaining gonocytes (8.20%) were aneuploid, from hypo-haploid to hypo-diploid R (Figure 8O), supposedly showing R genome elimination. In 3 individuals we found 7 gonocytes (11.48%) with 1-2 micronuclei without the RrS1 signal and 10–12 signals in the main nuclei (Figures 7E, 8L) (Supplementary Table S7). These results suggest that gonocytes in RRL tadpoles eliminate L genome, which is consistent with the results from metaphase plates.

#### Lack of a spatial separation between *P. ridibundus* and *P. lessonae* genomes

Visual analysis of 581 interphase G cells from tadpoles of all genotypes revealed random distribution of RrS1 signals within the area of cell nuclei and lack of peripheral chromatin compartments containing signals or devoid of them.

### Chromosome number and composition of SSCs, spermatocytes and spermatozoa in adult males. Genome transmission and fertility of males evidenced by crossing experiments

#### Diploid RL males

Eleven males aged 2–6 years were investigated. We analyzed 225 metaphase plates for all of them (## 1–11), and 148 interphase nuclei for 8 individuals (## 4–11) (Supplementary Tables S8, S9).

**FIGURE 9:**
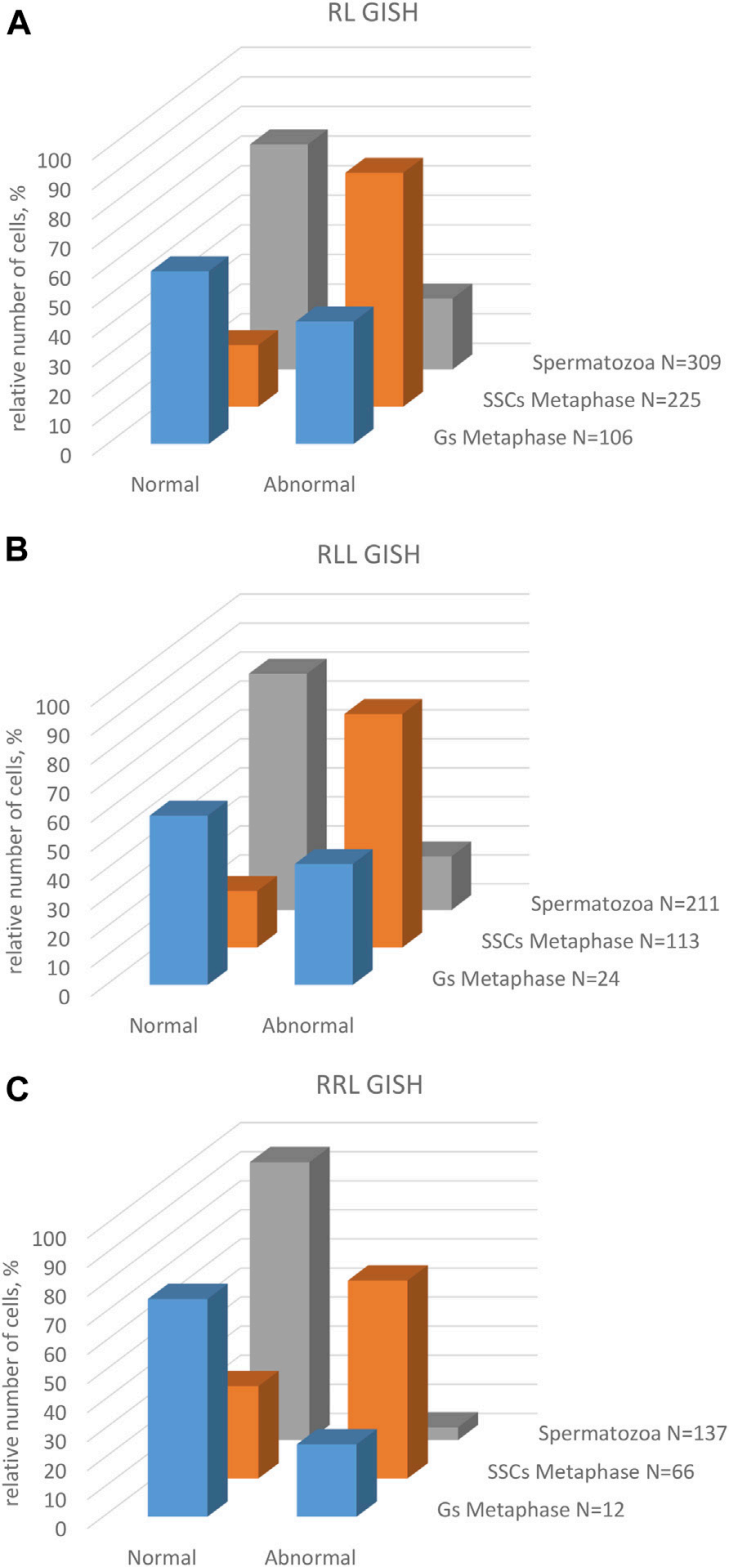
Frequency of normal and abnormal germ line cells in pre-spermatogenesis and active spermatogenesis. Relative number of cells in two groups highlighted on the basis of their normal or abnormal genomic composition. Gs and SSCs represent data from metaphase plates, spermatozoa assessed on the same chromosomal preparations after GISH/FISH hybridization. Graphs represent diploid RL males **(A)** or triploid RLL **(B)** and RRL **(C)** males.

**FIGURE 10:**
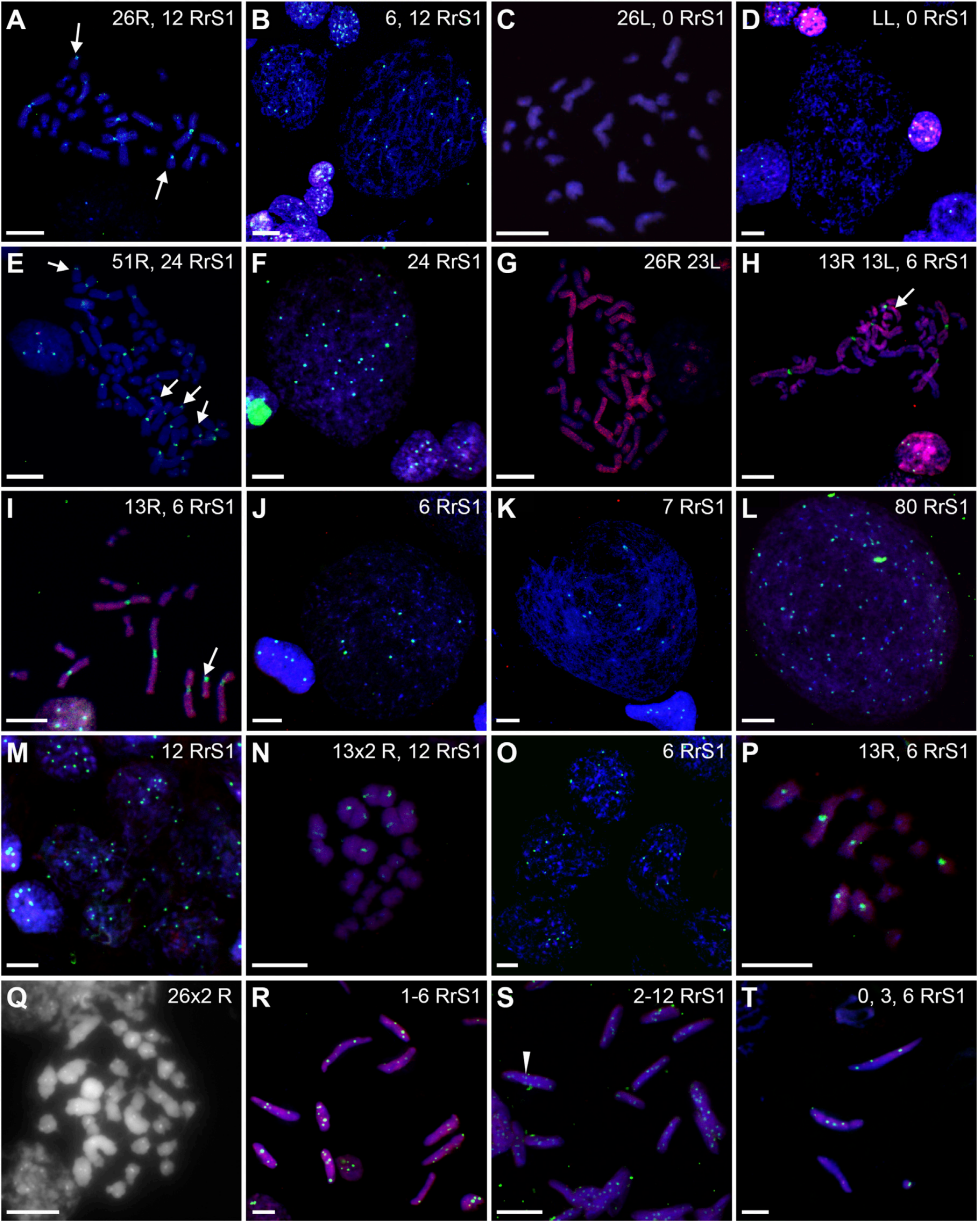
Genomic status of germ line cells in gonads of diploid RL adult males. Chromosomal preparations from gonadal homogenates were probed with whole genomic *P. ridibundus* probe Cy5 (red) **(B,D–K,M–P,R–T)**, or *P. lessonae* probe Cy5 (red, **A,C**) and RrS1 *P. ridibundus* pericentromeric probe labelled with FITC (green) **(A–F,H–P,R–T)** or with Cy5 (red) **(C)**, or without centromeric probe **(G)**. DNA stained with DAPI (blue). **(Q)** AMD-DAPI staining. Somatic cells display 5-6 RrS1 signals **(B,D,E,F,H,J,K,M)**. Arrow - medium-small heterobranchial submetacentric chromosome (8) with centromeric RrS1 signal. **(A)** metaphase 2n RR with 12 RrS1 signals, note 2 small chromosomes with signals, #11. **(B)** Interphase SSCs with 6 and 12 RrS1, #4. **(C)** Metaphase 2n L, 53, R+RrS1 green, L red. **(D)** Interphase LL, no RrS1 signal, #4. **(E)** 4n R metaphase, note 24 RrS1 signals, #6, RrS1 probe (green), LL probe (red). **(F)** Polyploid 4n interphase with 24 RrS1 signals, #4. **(G)** 4n RL tetraploid metaphase, only R probe, #8. **(H)** Metaphase with 26 chromosomes, 13R and 13L, note 6 RrS1 signals, #10. **(I)** Metaphase with 13R chromosomes, note 6 strong RrS1 signals, #11. **(J)** interphase nucleus, note 6 RrS1 signals, #10. **(K)** large interphase nucleus Ø 70 µm, note seven RrS1 signals, #10. **(L)** Polyploid interphase with 80RrS1 signals Ø 70µm, #11. **(M)** secondary spermatogonia in cyst, showing 12 RrS1 signals, #9. **(N)** meiotic metaphase I, regular 13 *P. ridibundus* bivalents with 2 × 6 RrS1 signals, #6. **(O)** Secondary spermatogonia in cyst, showing 6 RrS1 signals, #10. **(P)** 13 *P. ridibundus* univalents with 6 RrS1 signals, #11. **(Q)** meiotic metaphase I with 26 × 2 R bivalents showing strong centromeric dots after AMD-DAPI staining, probably precursor of diploid R sperms, #7. **(R)** Spermatozoa with 1-6 RrS1 signals, #10. **(S)** Spermatozoa with 2-12 RrS1 signals, #6. **(T)** Spermatozoa with 0, 3 or 5 RrS1 signals, #10. White arrowhead—spermatozoa with 12 RrS1 signals. Scale bar 10 µm.

**FIGURE 11:**
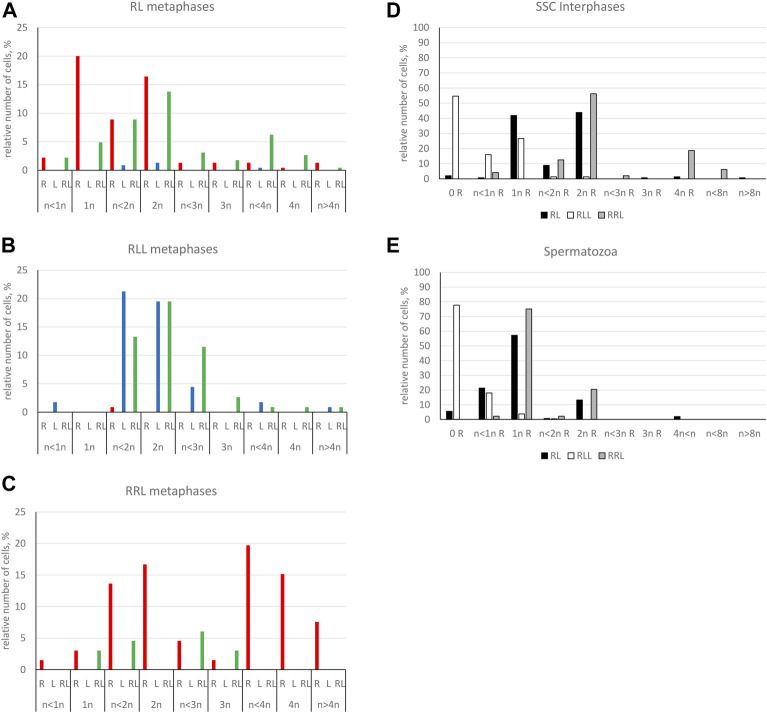
Genomic composition of spermatogenic cells during active spermatogenesis. Relative number of cells displaying various genomic compositions according to GISH/FISH analysis of chromosomal spreads. **(A)** RL metaphase plates. **(B)** RLL metaphase plates. **(C)** RRL metaphase plates. **(D)** Interphase SSCs in 2n and 3n males. **(E)** Spermatozoa in 2n and 3n males.

Only 20.89% of SSCs metaphases were regular (Figure 9A), of which 16.44% were diploid RR (Figures 10A, 11A), 1.33% were diploid LL (Figure 10C), 0.45% were tetraploid RRRR (Figure 10E) and 2.67% were tetraploid RRLL (Figure 10G). The remaining 79.11% were abnormal (Figure 9A), of which 37.77% contained only one type of chromosomes (20% haploid R, Figure 10I, 16.43% aneuploid R from hypo-haploid to more than tetraploid, and 1.34% aneuploid L) and 41.34% had mixed R + L chromosomes (13.78% diploid, Figure 10H, 27.56% from hypo-haploid to more than octoploid).

Among interphase nuclei of SSCs, 43.9% were RR (10–12 RrS1 signals) (Figures 10B, 11D), and 1.34% were RRRR (24 RrS1 signals) (Figure 10F). Next, 2.01% cells contained only L genome (no RrS1 signal) (Figure 10D), while 41.87% had R or mixed R+L composition (5-6 RrS1 signals) (Figures 10B,J). The remaining nuclei (10.88%) were aneuploid (Figures 10K,L), from hypo-haploid R to more than octoploid R. Some interphase cells were extremely large with different number of RrS1 signals varying from 7 (Figure 10K) to 80 (Figure 10L). These cells may represent the large SSC seen in histological sections, and should be assigned as polyploid for L or R genome, or both genomes.

We observed nests of secondary spermatogonia with RR genomes (12 RrS1 signals) in male #9 (Figure 10M, Table 1), and R genome (5-6 RrS1 signals) in male #10 (Figure 10O). Haploid secondary spermatogonia were probably the descendants of haploid SSCs, and they would probably form univalents. In two males (## 2 and 4), no meiotic cells were found. In most of males (## 3, 5, 6, 7, 8, 9, 10, 11) spermatocytes had 13R bivalents (Figure 10N), and in two (## 6 and 7) also 26R bivalents (Figure 10Q). Three males (## 1, 3 and 9) had 13L bivalents. Two males (## 3 and 9) had 13R bivalents and 13L bivalents, which suggested the production of two types of spermatozoa (L and R, see Table 1). We also found 13L univalents in male #1, and 13R univalents in males #5 and #11 (Figure 10P). Spermatocytes with 26 bivalents (both 13R and 13L bivalents) were present in male #8, which proved the possibility of producing RL sperm.

**TABLE 1:** Consistency between the genomic composition of SSCs, spermatocytes and spermatozoa in individual males. Data represent results of GISH/FISH assessment of chromosomal plates and interphases, additionally confirmed in AMD/DAPI assessment of chromosomal plates. Genomic compositions in spermatocytes are depicted as RR or LL for 13 bivalents, RRRR for 26 bivalents, RL for 13 R univalents and 13 L univalents. Letters in different colors denote genomic compositions ensuring proper meiosis (SSCs) or full ploidy of gamete compositions: blue – *P. lessonae* genome, red – *P. ridibundus* genome, green – hybrid genomic composition.

**Genotype**							
**No**	**No of male**	**population type**	**somatic**	**SSCs metaphases and interphases**	**Secondary spermatogonia/spermatocytes**	**Spermatozoa in tubules**	**transmitted to progeny^1^**
	**RL**						
1	44/16	R–E–L	RL	R, LL^2^	L^2^ LL^2^	L^2^	L
2	45/16	R–E–L	RL	R, LL^2^, RL, RRLL, R*, L*^2^, RL*		L^2^	infertile
3	47/15	L–E	RL	RR^3^, LL^2^ ^,^ ^3^, RRLL^3^, R*^3^, RL*^3^	RR^2^, LL^2^	R^2^, L^2^	–
4	47/16	R–E–L	RL	R^3^, RR^3^, RRR, RRRR^3^ ^,^ ^4^, RL, R*^3^, RL*	R, RR, RRRR^2^, R*,	R, RR	infertile
5	53/16	L–E	RL	R, RR^3^, LL, R*, RL*	R^2^, RR^2^	R, L	R
6	57/16	R–E–L	RL	R, RR^3^, RRR, RRRR, RRLL^2^, R*, L*, RL*	RR^2^, RRRR^2^	R, RR	R
7	59/16	R–E–L	RL	R^**3**^, RR^3^, R*	RR^2^, RRRR^2^	R, L, RR, R*	–
8	62/16	E–E	RL	RR^2^ ^,^ ^4^, RL, RRLL^3^, RL*	RR^2^, RRLL^2^	R, RR, R*	–
9	65/16	L–E	RL	R^3^ ^,^ ^4^, RR^3^, L^4^, LL^2^ ^,^ ^3^, RL^3^, L*, RL*	R, RR^3^, LL^2^, R*	R, L, R*	–
10	67/16	L–E	RL	R^4^, RR^3^, LL, RL, RRLL, RL*	R, RR^2^, R*	R, L, R*	–
11	75/16	R–E–L	RL	R^3^, RR^3^, R*^3^, RL*	RR^2^	–	–
	**RLL**						
12	2/3n/15 Z5	E–E	RLL	R^4^, RR^4^, L^4^, LL^2^, RL, RRLL, RLL*^2^, RL*	L, LL, RL, RL*	R, L, R*	L
13	10/3n/15 Z5	E–E	RLL	R^4^, L^2^, LL^3^, L*, R*^4^,	LL	L	L
14	25/16	E–E	RLL	LL^3^, RL, RLL, L*, R^4^, R*^4^, RL*	L^2^, LL^2^	R, L, R*	–
15	40/16	E–E	RLL	L^2^, LL^3^, RL, L*, R*, RL*	LL^**2**^	L	L
16	63/16	E–E	RLL	LL, L*, RL*		L	–
	**RRL**						
17	3/3n/15 B7	E–E	RRL	R, RR^3^, RRRR^3^, R*	R, RR, R*	R, RR, R*	R
18	10/3n/15 B8	E–E	RRL	RR^3^, RRRR^4^, R*	R, RR, RRRR, R*	R, RR	R
19	14/16	E–E	RRL	R, RRRR^3^ ^,^ ^**4**^, RRLL^2^, R*, RL*	RRRR^2^	R, RR	–

1 according to crossing experiments

2 assessed in AMD-DAPI staining only, without FISH for *ridibundus* centromeric sequence

3 assessed in GISH and additionally in AMD-DAPI staining

4 assessed in interphases only

L–in spermatozoa means cells without R signal (RrS1 either AMD-DAPI), R* – aneuploid; hypo– or hyperploid R (no L admixture), L*–aneuploid; hypo– or hyperploid L (no R admixture), RL*–aneuploid R+L compositions of variable ploidy level; various numbers of mixed L and R chromosomes.

Among 309 spermatozoa analyzed in males ##4-10, (Supplementary Table S10), 57.27% were haploid R (Figures 10R,S, 11E) and 13.27% were diploid RR (Figure 10S). Only 5.5% had no RrS1 signal (Figure 10T), which indicated the presence of L genome. We also observed aneuploid spermatozoa (Figure 9A), 21.36% were hypo-haploid R (Figures 10R,T) and 2.6% hypo-diploid or more than tetraploid R.

**FIGURE 12:**
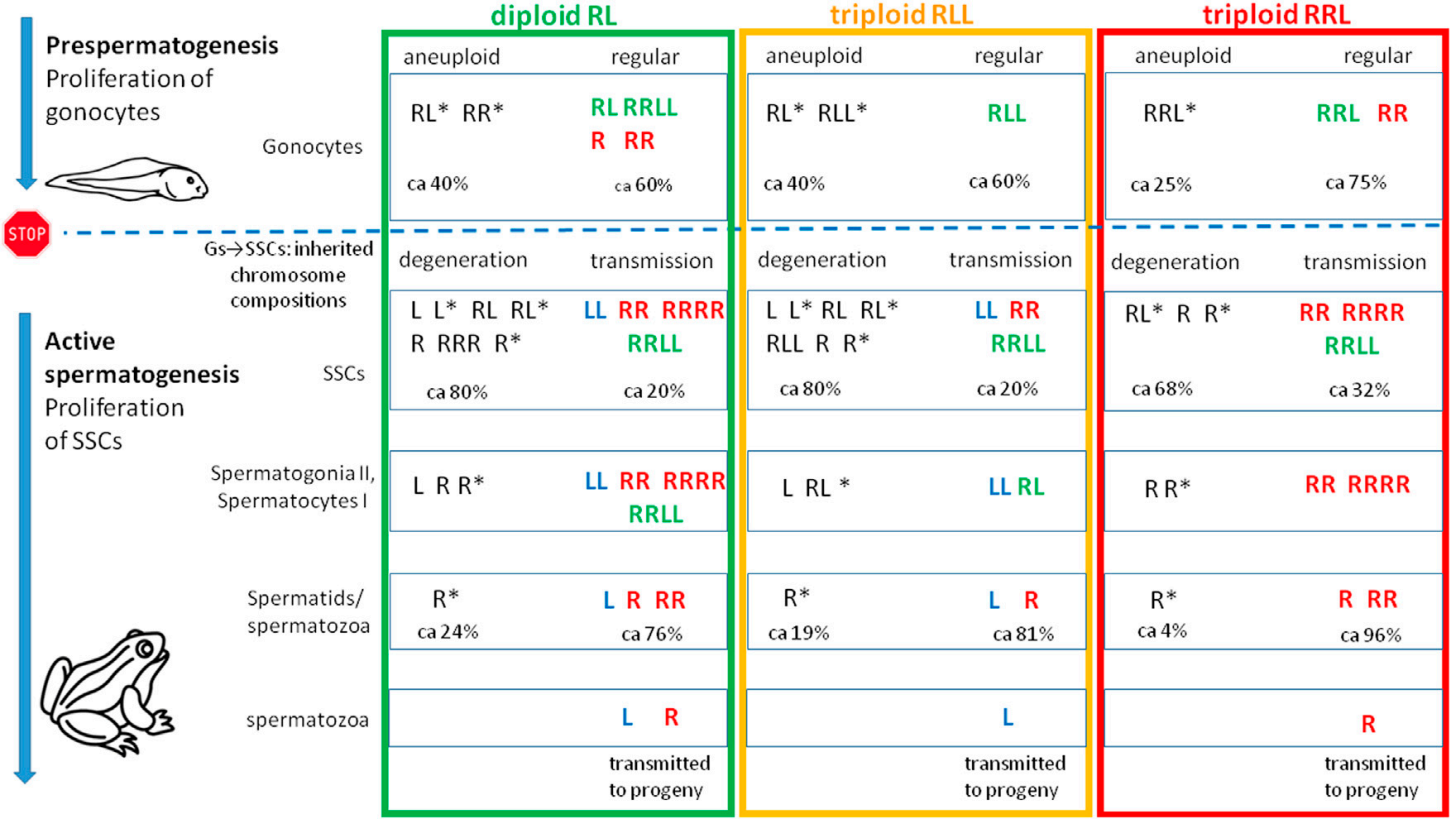
Tracing variability of genomic compositions during male ontogeny in diploid RL and triploid RLL and RRL *P. esculentus* hybrid males. The diagram illustrates qualitative description of genomic states found in metaphase and interphase cells (based on Table 1), beginning from gonocytes (Gs) in tadpoles at the stage of prespermatogenesis, then during active spermatogenesis in consecutive cellular generations including spermatogonial stem cells (SSCs), secondary spermatogonia and spermatocytes in nests, and finally in spermatozoa. STOP sign and a dashed blue line denotes the period following prespermatogenesis in juvenile males when gonocytes become dormant and cease the mitotic activity. After reaching sexual maturity SSCs begin their mitotic cycles and differentiate into secondary spermatogonia. Spermatogenic cells possessing either regular or aneuploid chromosomal compositions are entering the next differentiation stage, but part of them degenerates. Still, some aneuploid spermatozoa are present in seminiferous tubules.

To investigate gamete contribution of each hybrid male into progeny, we performed crosses of 5 diploid males (## 1, 2, 4, 5 and 6) with *P. lessonae* or *P. ridibundus* females (Supplementary Tables S11, S12). Among five diploid males examined, three gave viable progeny while two (#2 and 4) were infertile. The structure of testis of sterile males exhibited numerous degenerations of the spermatogenic cells, especially spermatocytes, which supposedly showed rejection of aneuploid cells. Despite we detected L and R spermatids in male #1, we obtained only *P. esculentus* tadpoles (N = 30) after crossing of diploid RL male with *P. ridibundus* female (Figure 12, Table 1). It suggests that R spermatozoa did not contribute to viable offspring. After crossing of male #5, producing L and R spermatids, with *P. ridibundus* female, we obtained *P. ridibundus* tadpoles (N = 30) suggesting that L spermatozoa did not contribute to viable offspring (Table 1). Male #6 had RR, RRRR and RRLL composition of SSCs and produced haploid and diploid R spermatids. After crossing this male with the *P. lessonae* female we obtained 30 *P. esculentus* tadpoles and we concluded that this male transmitted R genome (Figure 12, Table 1). Using PCR test we were not able to confirm ploidy of the tadpoles, thus we do not know whether diploid RR spermatozoa resulted in triploid progeny.

#### Triploid RLL males

We analyzed 263 SSCs (113 metaphase chromosomal plates and 150 interphase nuclei) obtained from 5 males (## 12–16) aged 4–6 years (Supplementary Tables S8, S9).

**FIGURE 13:**
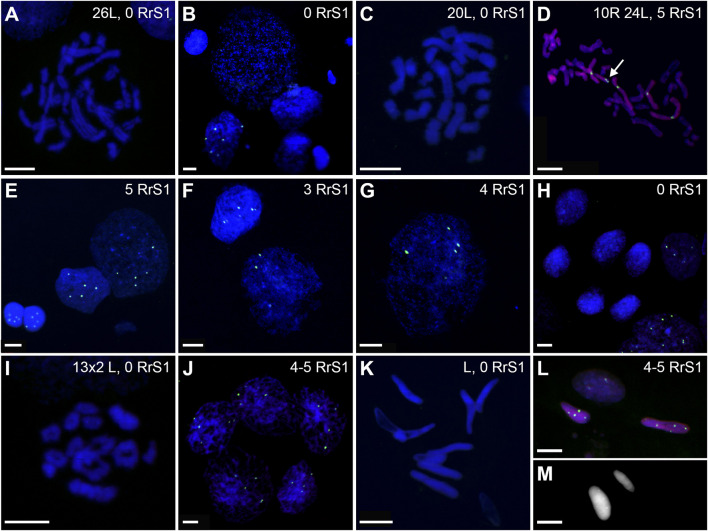
Genomic status of germ line cells in gonads of triploid RLL adult males. Chromosomal preparations from gonadal homogenates were probed **(A–L)** with the whole genomic *P. ridibundus* probe Cy5 (red) and RrS1 *P. ridibundus* pericentromeric probe labelled with FITC (green), DNA stained with DAPI (blue). **(M)** AMD-DAPI staining. Somatic cells display 5-6 RrS1 signals **(B,E,H,J)**. Arrow - medium-small heterobranchial submetacentric chromosome (8) with centromeric RrS1 signal. **(A)** mitotic prophase with 26L chromosomes, note a lack of RrS1 signals, #13. **(B)** interphase of SSC with 0 RrS1, note 6 signals in somatic cell, #13. **(C)** aneuploid L metaphase with 20 chromosomes, #13. **(D)** aneuploid metaphase with 10R and 24L chromosomes, note 5 RrS1 signals, #12 **(E)** two interphase SSC with 5 RrS1 signals without R genome elimination, two small cells are spermatids with 6 RrS1 signals, #13. **(F)** interphase SSC with 3 RrS1 signals with incomplete R genome elimination, #12. **(G)** large interphase SSC Ø 50µm, with incomplete R genome elimination, note 4 RrS1 signals, #12. **(H)** meiotic prophase I at zygotene stage in 6 nuclei bearing L genome, without RrS1 signals, #13. **(I)** meiotic metaphase I with 13L bivalents, male 13, #13. **(J)** meiotic prophase I at zygotene stage in 5 nuclei with 4-5 RrS1 signals, evidencing no R genome elimination, #12. (K) L spermatozoa, #13. **(L)** spermatozoa with 4-5 RrS1 signals, #12. **(M)** small spermatozoa without R dots and big spermatozoa with R dots after AMD-DAPI staining revealing *P. ridibundus* centromeric regions, #14. Scale bar 10 µm.

Only 19.49% of metaphases were regular LL (Figure 9B, 11B, 13A), suggesting the production of L spermatozoa. The remaining 80.51% were abnormal (Figure 9B), of which 30.08% were aneuploid L, mostly hypo-diploid (Figure 13C), up to more than tetraploid and one cell (0.88%) was hypo-diploid R, 49.55% were mixed R + L, 13.27% hypo-diploid, 19.49% diploid, 2.65% triploid other than RLL, and 14.14% from hypo-triploid (Figure 13D) to more than tetraploid.

Among the SSCs interphase nuclei, 54.67% have L or LL genomes (no RrS1 signal) (Figures 11D, 13B) similarly to mitotic chromosomal spreads. 26.67% and 1.33% of cells represented R (or RL) (5-6 RrS1 signals, Figure 13E) and RR (or RRLL) (10-12 signals) genomes respectively. The remaining nuclei were aneuploid and had various numbers of RrS1 signals: hypo-haploid (16%, Figure 13F) and hypo-diploid (1.33%). Some aneuploid cells had extremely big nuclei with incomplete removal of R genome (Figure 13G). They could represent polyploid cells with endoreplicated L genome, and may correspond to abnormal degenerating SSC detected in histological sections.

In four males (## 12, 13, 14 and 15), spermatocytes had 13L bivalents (Table 1). Male #12 had also low number of 13L univalents or 13 bivalents with 11–12L pairs and 1–2R pairs. After GISH/FISH labelling we found cysts of spermatocytes at zygotene stage usually having L genome (no RrS1 signals) (Figure 13H) but in male #12 some spermatocytes had R genome (4-5 RrS1 signals) (Figure 13J). This male had chromosomal plates with 13L univalents or 13L bivalents, as well as mixed R+L univalents, 13R univalents and 13L univalents, and aneuploid plates. The male #13 had only L bivalents, 5 of 13L bivalents (Figure 13I) and one aneuploid of 11L bivalents.

Among 309 spermatozoa examined, the majority (77.75%) had L or LL genomes (no RrS1 signal) (Figures 11E, 13K) (Supplementary Table S10), 3.76% have haploid R genome (Figures 13L). 18.49% spermatozoa were aneuploid (hypo-haploid R, Figure 13L, and hypo-diploid R) (Figure 9B). Spermatozoa bearing R genome were bigger than spermatozoa with L genome (Figure 13M) suggesting the formation of sperm with haploid L, but also haploid R and diploid RL genomes as well as aneuploid ones.

Gamete contribution of each hybrid male into progeny was assessed after crosses of three RLL males (##12, 13 and 15) with *P. ridibundus* females (Supplementary Tables S11, S12). All analyzed tadpoles from three crosses (number of tadpoles: 30, 25 and 26 respectively) were *P. esculentus*. Despite male #12 had LL, RR and RRLL genome composition of SSCs and spermatozoa with R and L genomes, it transmitted only L genome to the viable progeny (Table 1). Males ##13 and 15 had LL genome in SSC, L genome in sperm and also transmitted L genome to the progeny (Figure 12, Table 1).

#### Triploid RRL males

In chromosomal slides from 3 males (## 17–19) aged 5–7 years, we examined 114 SSCs, 66 metaphases and 48 interphases (Supplementary Tables S8, S9).

**FIGURE 14:**
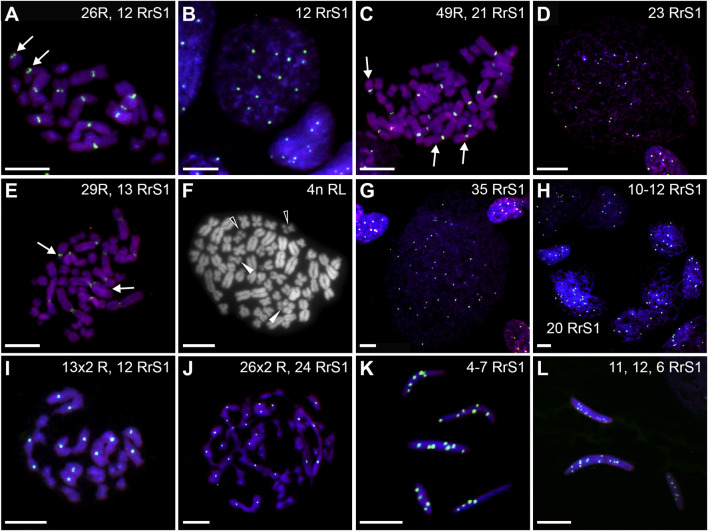
Genomic status of germ line cells in gonads of triploid RRL adult males. Chromosomal preparations from gonadal homogenates were probed **(A–E,G–L)** with the whole genomic *P. ridibundus* probe Cy5 (red) and RrS1 *P. ridibundus* pericentromeric probe labelled with FITC (green), DNA stained with DAPI (blue). **(F)** AMD-DAPI staining. Somatic cells display 10-12 RrS1 signals **(B,D–G,J)**. Arrow - medium-small heterobranchial submetacentric chromosome (8) with centromeric RrS1 signal. Chromosomal preparations from gonadal homogenates were probed **(A–E, G–L)** with the whole genomic *P. ridibundus* probe Cy5 (red) and RrS1 *P. ridibundus* pericentromeric probe labelled with FITC (green), DNA stained with DAPI (blue). **(F)** AMD-DAPI staining. Somatic cells display 10-12 RrS1 signals **(B,D–G,J)**. Arrow - medium-small heterobranchial submetacentric chromosome (8) with centromeric RrS1 signal. **(A)** mitotic metaphase with 26R chromosomes and 12 RrS1 signals, after L elimination, #18. **(B)** interphase of SSC with 12 RrS1, note 10 signals in somatic cell, #18. **(C)** mitotic metaphase with 49R chromosomes and 21 RrS1 signals, showing endoreplication event, #19. **(D)** interphase SSC after endoreplication, note 23 RrS1 signals, #19. **(E)** mitotic metaphase with 29R chromosomes and 13 RrS1 signals, with incomplete R genome endoreplication, #19. **(F)** Tetraploid mitotic metaphase with mixed 50R+L chromosomes, note 4 chromosomes number 10 bearing NOR regions on their p arm, 2 of them with R centromeric signal (white solid arrowhead, R chromosomes) and 2 without the signal (white empty arrowhead, L chromosomes), #19. **(G)** giant interphase with 35 RrS1 signals, Ø 90µm, showing abnormally elevated genome endoreplication, #19. **(H)** meiotis prophase I with R zygotene complements bearing 10-12 or 20 RrS1 signals, #18. **(I)** meiotic metaphase I with 13R bivalents, #18. **(J)** meiotic metaphase I with 26 pairs of R bivalents and 24 RrS1 signals, #18. **(K)** R spermatozoa with 4-7 RrS1 signals, 4 or seven signals might be aneuploid ones, #17. **(L)** R spermatozoa with 11, 12 or 6 RrS1 signals, #18. Scale bar 10 µm.

Regular R metaphases only accounted for 31.84% chromosomal plates, among which 16.69% were diploid RR (Figures 9C, 11C, 14A) and 15.15% tetraploid RRRR (Figure 14C). The remaining 68.16% were abnormal (Figure 9C), bearing either only R chromosomes (51.51%, haploid R, hypo-haploid R, hypo-diploid R and up to tetraploid (Figure 14E), or more than tetraploid R) or mixed R+L sets (16.65%, from haploid up to triploid). Additional AMD-DAPI staining in male #19 showed also 4n RL metaphases (Figure 14F).

More than a half of interphase nuclei (56.27%) had 10-12 RrS1 signals (Figures 11D,14B), which indicated the presence of 2 R chromosome sets (RR or RRL), possibly precursors of haploid R sperm. Next 18.75% of cells were tetraploid RRRR with 20–24 RrS1 signals (Figure 14D), probably giving rise to diploid RR sperms. The remaining nuclei (24.98%) were aneuploid and had various numbers of RrS1 signals (from hypo-haploid to more than tetraploid, mostly hypo-diploid). Some nuclei were very large and showed as much as 35 RrS1 signals, suggesting at least two rounds of genome endoreplication (Figure 14G).

Bivalents and univalents analyzed after AMD/DAPI staining showed 13R bivalents in males ##17 and 18, and also 26R bivalents in male number 19 (Table 1). Additional analysis after GISH/FISH labelling with the whole-genome R and RrS1 probes (males ## 17 and 18) revealed diploid nuclei with 10–12 RrS1 signals (Figure 14H) in nests of meiotic spermatocytes at zygotene. We confirmed the presence of only R chromosomes in 19 meiotic chromosomal plates, where 12 spermatocytes had 13R bivalents (Figure 14I), 3 had 26 bivalents (Figure 14J) and 4 were aneuploid below 13 or 26 bivalents. These results suggested the transmission of haploid or diploid R chromosome sets into spermatozoa.

We examined 137 spermatozoa (Supplementary Table S10). The vast majority of spermatozoa (95.62%) were regular, including 75.18% haploid R cells with 5-6 RrS1 signals (Figures 9C, 11E, 14K,L), and 20.44% diploid RR cells with 10–12 RrS1 signals (Figure 14L). The remaining 4.38% aneuploid spermatozoa were hypo-haploid R or hypo-diploid R (Figure 14K, 9C). These results show that RRL males produced mostly haploid R, but also diploid RR and aneuploid spermatozoa.

To investigate gamete contribution of RRL hybrid males into progeny, we performed crosses of 2 triploid RRL males (## 17, 18) (Supplementary Tables S11, S12). Male # 17 produced 26 *P. ridibundus* tadpoles in mating with *P. ridibundus* female. Crossing of male # 18 with the *P. lessonae* female resulted in 26 *P. esculentus* tadpoles. Both RRL males (## 17 and 18) had RR or RRRR chromosomal sets in their SSCs, produced haploid R and diploid RR spermatozoa and transmitted R genome to their progeny (Figure 12, Table 1).

#### Lack of micronuclei in SSCs

Unlike tadpoles, which had micronuclei in their interphase Gs on chromosomal spreads, interphase SSCs did not have any micronuclei when analyzed in chromosomal spreads of RL, RLL and RRL adult males, indicating no chromosome elimination in adults, which is in line with the results of histology.

## Discussion

We analyzed the entire process of spermatogenesis in hybrid water frogs, from undifferentiated gonads through the time when tadpole gonads sexually differentiate into testes and further to completion of metamorphosis and then in sexually mature males. We have obtained evidence that chromosomes are eliminated and reduplicated only in gonocytes during early gonad development in tadpoles (prespermatogenesis) but not in SSCs in adults (active spermatogenesis).

We obtained consistent results from three different approaches. First, development of gonads studied in histology revealed no differences in morphology of male gonads and germ cells in both diploid and triploid individuals. We confirmed the presence of micronuclei only in gonocytes, but not in SSCs, which means that genome rearrangements (elimination and reduplication of chromosomes) take place during prespermatogenesis, but not during active spermatogenesis in adult males. Secondly, we studied genome composition and ploidy level in the same classes of spermatogenetic cells in various individuals in tadpoles and in adults. Thirdly, we traced genomic composition of SSCs, spermatocytes and spermatozoa in individual adult males that were crossed with females of the parental species and gave progeny. In this way, we were able to estimate the efficiency of spermatogenesis and genome transmission to effective spermatozoa.

Here, for the first time we used GISH with the whole genome *P. ridibundus* probe combined with FISH to *P. ridibundus* specific RrS1 pericentromeric repeat (Ragghianti et al., 1995; Marracci et al., 2011) to assess genome composition and ploidy level in various spermatogenetic cell lines in testes. Earlier, GISH and CGH were successfully applied on meiotic and mitotic chromosomes of interspecific hybrids from polyploid *Ambystoma* and diploid *Pelophylax esculentus* (Bi and Bogart, 2006; Zaleśna et al., 2011; Doležálková et al., 2016). Another method, which allows to distinguish *P. ridibundus* chromosomes, is FISH with the probe to RrS1. This repetitive sequence was observed in the pericentromeric region of 5 large and one heterobranchial medium-small chromosome (no. 8) (Ragghianti et al., 1995, 2004), while others reported its presence in all 13 chromosomes of *P. ridibundnus*, but with different intensity of signals (7 chromosomes exhibit strong signals, 6 chromosomes exhibit weak signal) (Marracci et al., 2011; Dedukh et al., 2020b; Dedukh et al., 2022b). Moreover, we detected signals in diploid and triploid *P. esculentus* in interphase nuclei of somatic and germ cells which significantly improved our analysis.

In cytogenetic analysis of prespermatogenesis in diploid and triploid tadpoles, we recorded the majority of germ cells before genome elimination, while the minority of cells were undergoing or finished elimination and endoreplicated their genome before the completion of metamorphosis. The majority of germ cells in RL and RRL individuals rejected the L genome, while RLL triploids preferentially removed R genome, which is consistent with the known models of hybridogenesis in water frogs (Christiansen et al., 2010). However, spermatogenic germ cells with genomic compositions ensuring successful meiosis consisted only a small pool in adult males (Figure 9), as opposed to the prevailing portion of aneuploid, mainly abnormal cells. As it turned out, even a small group of regular SSCs contributed to the formation of spermatozoa with the correct genomic compositions ensuring fertility of hybrid males.

### Spermatogenesis in hybridogenetic frogs and micronuclei as a marker of prespermatogenesis

In this and the former study (Haczkiewicz et al., 2017), we have shown that spermatogenesis in water frogs has similar stages as in mammals, thus we were using the same terminology for these two diverse vertebrate groups. Spermatogenesis in water frogs is divided into two main steps: prespermatogenesis in tadpoles until the completion of metamorphosis, and active spermatogenesis that starts at sexual maturation and lasts during the whole adult life of a male. However, these stages differed by various types of germ cells, as gonocytes (Gs) were observed during prespermatogenesis while spermatogonial stem cells (SSCs) were present during active spermatogenesis of adult males. A hallmark of Gs in hybridogenetic frogs is the formation of micronuclei that contain the eliminated chromosomes, which were eventually degraded by nucleophagy (Chmielewska et al., 2018; Dedukh et al., 2022b). Micronuclei formation is ceased when prespermatogenesis ends, which approximately coincides with the completion of metamorphosis. Since then, G cells transformed into dormant SSCs [or nascent spermatogonia, according to Pui and Saga (Pui and Saga, 2017)]. However, in a hybrid *P. esculentus* we previously noticed prolonged gonocyte proliferation, lasting in some individuals until the sexual maturation after third or even fourth hibernation [(Bartmańska and Ogielska, 1999), unpublished data Haczkiewicz], suggesting the possibility of genome elimination in juvenile males (i.e., 1- and 2-years old). During the initiation of sexual maturity (in 2- and mainly 3-years old males), the dormant spermatogonia activate and periodically renew the pool of SSCs or differentiate and enter meiosis. The previous studies focused on spermatogenesis in adult males (Günther, 1975; Heppich et al., 1982; Bucci et al., 1990; Doležálková et al., 2016) or juvenile *P. esculentus* (Ragghianti et al., 2007) because their authors erroneously considered that males have only one type of “primary spermatogonia” throughout their whole life. As we reported here, only Gs in hybridogenetic frogs eliminate genome of one of the parental species by forming micronuclei and reduplicate the remaining set of chromosomes, whereas SSCs do not produce micronuclei and therefore have no ability to eliminate chromosomes. Thus, micronuclei in hybridogenetic male water frogs are natural markers of prespermatogenesis, but not of active spermatogenesis. It suggests, that in SSCs of adult males there is no genome elimination but only its outcomes.

The development of testes in anuran amphibians was reviewed by Ogielska and Bartmańska (Ogielska and Bartmańska, 2009) and Roco et al. (Roco et al., 2021), including the parental species *P. lessonae* and *P. ridibundus* (Haczkiewicz and Ogielska, 2013; Haczkiewicz et al., 2017). Now we show that in both diploid and triploid *P. esculentus* the development and differentiation of testes is impaired and seminiferous cords in tadpoles and tubules in adults contain fewer germ cells in comparison to the parental species. Inaccurate genome elimination in gonocytes during early gametogenesis may cause cellular abnormalities leading to apoptosis, as we previously showed for *P. esculentus* male and female gonads (Szydłowski et al., 2016; Chmielewska et al., 2018; Dedukh et al., 2019). During active spermatogenesis in adult hybrid males, both SSCs and spermatocytes undergo degeneration via apoptosis detected by nuclear pycnosis and active caspase-3 signal. Such cells are detached from tubule wall into the lumen causing irregular tubule structure and empty spaces or almost sterile tubules in some males. Apart from the lack of germ cells, somatic cells are normally differentiated and active in regulation of reproductive behavior, therefore even sterile males show normal mating behavior.

### Elimination and endoreplication of the genome - outside the rules. Timing of both processes

Premeiotic endoreplication of a genome before meiosis is known in a variety of hybrid organisms, both animals (Stenberg and Saura, 2013) and plants (De Storme and Geelen, 2013). Now we demonstrated that genome elimination and endoreplication in *P. esculentus* are confined to the period of gonocyte mitotic activity in prespermatogenesis but these processes are not completed prior to metamorphosis in majority of gonocytes. This suggests possible continuation of genome elimination and endoreplication in juvenile males. We observed different genotype composition in diploid hybrids (namely, RL, R, RRLL, RR and significant numbers of aneuploids), therefore we assume that different stages of genome elimination are present in various cells. These may be: no elimination and no endoreplication, elimination without endoreplication, endoreplication without elimination, correct elimination and endoreplication, and incorrect elimination and/or endoreplication, respectively. In triploid individuals (both RRL and LLR) the majority of cells eliminated single copied genome from Gs. However, in other Gs we observed interphase nuclei with ploidy level above 4n suggesting the presence of several rounds of endoreplication. In RRL tadpoles, we found few interphase cells without the correct number of 12 RrS1 probe signals suggesting the extrusion of R genome. This finding certainly does not result from the problems with probe hybridization as there were other interphase cells, both gonocytes and somatic, with the fluorescent probe signal present on the same slides. This shows variations from the classical model of triploid hybridogenesis, which assumes that the haploid genome is eliminated, while the double-copy genome no longer needs to be duplicated (Christiansen, 2005, 2009; Christiansen et al., 2005, 2010; Christiansen and Reyer, 2009; Arioli, Jakob and Reyer, 2010). Similarly, the analysis of diplotene oocytes in triploid *P. esculentus* females also shows elimination of double copied genome and endoreplication of unreduced chromosomal sets (Dedukh et al., 2015; Dedukh et al., 2017). In diploids, elimination of L genome was reported by several authors (Uzzell, Hotz and Berger, 1980; Tunner and Heppich, 1981; Heppich et al., 1982; Uzzell et al., 1980; Tunner and Heppich 1981; Heppich et al., 1982) and of R genome by Vinogradov et al. (Vinogradov et al., 1990). Here, we demonstrate that genome elimination and endoreplication in diploid hybrids and genome elimination in triploid hybrids act normally in G cells during early gametogenesis. However, frequently observed alterations from the proper ploidy level suggest that these processes are not always separate and accurate.

Our previous results suggest that genome elimination is a gradual and multistep process including budding of individual chromosomes during interphase, as well as chromosomal misalignment followed by their lagging (Ogielska, 1994; Chmielewska et al., 2018; Dedukh et al., 2019; Dedukh et al., 2020b). Thus, the germ cells would have to proliferate with a chromosome number different from 2n or 3n in case of diploid and triploid hybrids, respectively. Indeed, in the tadpole testes studied herein, aneuploid metaphase plates were observed relatively frequently. Nevertheless, it is difficult to unambiguously demonstrate whether such aneuploid cells are transient before elimination completion, or lead to the formation of aneuploid SSCs in adult animals and thus to the formation of abnormal gametes. Alternatively, frequently found aneuploid SSCs might arise from chromosomal abnormalities *de novo* during SSCs’ mitotic cycles, but this is not very likely, as we detect ploidy variation in SSCs. Nevertheless, we showed that at least some aneuploid gonocytes survived to adulthood and transformed into SSCs. Such aneuploid SSCs were detected in adult testes, and thus probably were leading to the formation of aneuploid spermatozoa, present in seminiferous tubules in males of all three genotypes. We and others have found mitoses with 13 chromosomes at metaphase and anaphase in adult and juvenile males [this study (Heppich et al., 1982; Doležálková et al., 2016)], and females (Tunner and Heppich-Tunner, 1991) as well as in tadpole gonads (this study). Moreover, aneuploid oogonia were frequently observed during early development in RL and RLL individuals (Tunner and Heppich-Tunner, 1991; Dedukh et al., 2020b). These results suggest that gonocytes can divide with aneuploid or haploid number of chromosomes.

Both male and female gonocytes are able to overcome different checkpoints and produce at least some gametes with properly eliminated and properly endoreplicated genomes. Moreover, in water frogs cell cycle and even meiotic checkpoint seems to be not very strict as aneuploid and mispaired chromosomes in meiosis are able to proceed at least to diplotene stage in case of females (Dedukh et al., 2015; Dedukh et al., 2017; Dedukh et al., 2019) and to aneuploid primary spermatocytes and gametes in case of males [this study (Doležálková et al., 2016; Pustovalova et al., 2022)]. In diploid and triploid gynogenetic fish females in *Cobitis taenia* complex as well as parthenogenetic geckos (Dedukh et al., 2021; Dedukh et al., 2022a), only a small fraction (1.5%–11%) of oogonia has correctly endoreplicated genome allowing them to achieve diplotene stage. Nevertheless, the majority of oogonia failed to proceed beyond pachytene due to errors in bivalent formation and inability to pass the cell cycle checkpoints. On the contrary, spermatocytes in male *Cobitis* hybrids may pass the pachytene checkpoint, but finally they fail to complete meiosis resulting in sterile males (Kuroda et al., 2019; Dedukh et al., 2020b).

### Micronuclei in chromosomal spreads—bonus from the study of interphase nuclei

Micronuclei are well-documented structures found in gonocytes of triploid and diploid hybridogenetic water frogs and include individual eliminated chromosomes (Ogielska, 1994; Dedukh et al., 2017; Dedukh et al., 2019; Dedukh et al., 2020b) which are subsequently degraded by autophagy (Chmielewska et al., 2018). Similar way of genome elimination via micronuclei formation was recently reported in hybridogenetic Australian carp gudgeons of the genus *Hypseleotris* (Majtánová et al., 2021). In diploid and triploid *P. esculentus* tadpoles, we found 6%–11% of interphase Gs containing micronuclei. Recently, in whole mount *P. esculentus* gonads micronuclei were found in 10%–30% of gonocytes (Dedukh et al., 2020b). It suggest that in chromosomal spreads we could have lost some micronuclei due to the cell membrane breakage and subsequent loss of some cytoplasm components from the cells. Micronuclei were considered to contain R chromosomes if they had a strong RrS1 signal. However, if they did not have this signal, we can expect either *P. lessonae* chromosomes or *P. ridibundus* ones without an intense RrS1 signal. In accordance with the hybridogenesis pattern in *P. esculentus* we found that micronuclei bearing RrS1 signal were most abundant in RLL gonocytes, while in RL and RRL micronuclei were mainly lacking the signal. This is in perfect agreement with the study of Dedukh et al. (2020b) who found preferential L genome exclusion in RL individuals and R genome removal in the micronuclei of RLL individuals. We usually observed 1–4 micronuclei in spreads of interphase cells suggesting that several chromosomes had to be eliminated simultaneously. Recent studies in *P. esculentus* (haploid chromosomal set n = 13) showed similar number of 1-5 micronuclei per one cell (Chmielewska et al., 2018; Dedukh et al., 2020b), and in *Hypseleotris* fish hybrids (haploid chromosomal set 22 ≤ n ≤ 24) authors found 1–7 micronuclei per cell (Majtánová et al., 2021). Micronuclei usually contain one chromosome [this study and (Dedukh et al., 2020b)], however we also detected several micronuclei with 2 signals and one with 4 signals in gonocytes of RLL hybrid. Similarly, using anti-centromere antibody, Dedukh et al. (Dedukh et al., 2020b) showed that the majority of micronuclei contained one centromere each, and only 4% of micronuclei were lacking the signal (probably acentric chromosome fragments) or showing 2-3 anti-centromeric fluorescent signals. As neither we, nor others never observed 13 micronuclei in one cell we cannot rule out that elimination lasts during several cell cycles. However, if we accept that some of the micronuclei may have already been degraded by nucleophagy, which is a rapidly occurring phenomenon (Rello-Varona et al., 2012), we can assume that we catch at the moment only intact nascent micronuclei. Nevertheless, the observation of hypo-diploid chromosomal sets in diploid tadpoles or hypo-triploid sets in triploid tadpoles, with lower number of R or L chromosomes suggests the graduality of the process. Together with the observation of 1-2 misaligned chromosomes during mitosis at metaphase stage (Ogielska, 1994; Dedukh et al., 2020b), suggesting that these chromosomes might be encapsulated as micronuclei in telophase, our results also confirm that elimination of full chromosomal set of one parental genome may take place in several cell cycles.

### Aneuploid and polyploid germ line cells present in tadpoles and adult males witness imprecise hybridogenesis

In all types of hybrids (RL, RLL and RRL), we found many aneuploid germ cells. In tadpoles, aneuploid Gs constituted less than 50% of cells, whereas in adults the number of aneuploid SSCs was about 80% (Figure 9). These results were consistent with the histological observations where we observed many abnormal and degenerating germ cells which we interpreted as resulting from incorrect chromosome elimination and/or reduplication, and failing to complete spermatogenesis.

We thus suggest that irregularities in genome elimination and endoreplication in Gs of tadpoles are transferred to the resulting SSCs and the gametes in adults. Similarly to our result, a mixture of cells of various ploidy and aneuploids, from less than 1C, 2-3C to 4C was observed in gonads of adult diploid and triploid RLL hybrid males from various population types (Vinogradov et al., 1991). Moreover, germ cells with RL and RLL genomes were scarce or absent at least in some diploid and triploid males, respectively (Vinogradov et al., 1990, 1991). Heppich et al. (1982) have found only R chromosomes in “primary spermatogonia” (i.e., SSCs) in testes of adult males, however they studied a limited number of individuals. The analysis of spermatogenesis of *P. esculentus* and *P. ridibundus* adult diploid males from Eastern Ukraine revealed numerous aneuploid mitotic SSCs metaphase plates with chromosome numbers ranging from below 13 (hypohaploid), above 13 (hyperhaploid), below 26 (hypodiploid) to above 26 (hyperdiploid), as well as 3n and 4n. The number of bivalents ranged from 13 univalents, 13 bivalents (normal) to 26 bivalents (Veregina et al., 2014). The abnormalities in both taxa were similar, but in *P. esculentus* they were more frequent (40% primary spermatocytes were normal) than in *P. ridibundus* (75% were normal). The authors hypothesized that aneuploid spermatocytes may result in aneuploid gametes (Veregina et al., 2014) and our results showing aneuploid spermatozoa confirmed their presumption. Also a study of Fedorova and Pustovalova (Fedorova and Pustovalova, 2021) on adult diploid *P. esculentus* males complement our research on spermatocytes and functional spermatozoa. They compared the number of meiotic chromosomes with sizes of spermatozoa and sperm productivity of the same males and found a variety of meiotic chromosomal plates in testes of adult diploid males, from all normal spermatocytes (fully fertile males) to none (sterile males) and with predominating aneuploid plates (decreased fertility) and 4n plates (prospective diploid sperm). However, when they compared the meiotic plates with urinary sperm head sizes they found no link between these two features. Their and our data clearly show that germ cell aneuploidy is common and that each male has his own unique features of spermatogenesis.

Not only spermatogenesis, but also oogenesis in *P. esculentus* yields in aneuploid gametes. Studies on lampbrush chromosomes of diplotene oocytes (Bucci et al., 1990; Dedukh et al., 2015; Dedukh et al., 2017; Dedukh et al., 2019) showed 13 bivalents of *P. ridibundus* (in RL and RRL hybrids) or *P. lessonae* (in LLR hybrids) as expected from the hybridogenetic model. However, various combinations of uni- and bivalents, as well as aneuploids were also detected. It was suggested, that such oocytes probably do not result in viable progeny as fertilization of aneuploid gametes must yield in abnormal embryos and high mortality (Christiansen et al., 2005; Christiansen, 2009). Such aneuploid embryos died during early ontogenesis due to arrested blastulae, exogastrulae, oedema (Ogielska, 1994; Christiansen, 2009).

We found that the great variety of gonocytes, SSCs, primary spermatocytes and spermatozoa sizes reflected their ploidy level. It is a well-known rule that cell size is correlated with DNA content (Gregory, 2001); for *P. esculentus* it works very well in erythrocytes when distinguishing diploid from triploid animals (Vinogradov et al., 1990; Ogielska, Kierzkowski and Rybacki, 2005) and in eggs when distinguishing haploid from diploid cells (Berger and Uzzell, 1977; Christiansen et al., 2005; Czarniewska et al., 2011). In histological sections of adult testes of all hybrid types, we observed cysts containing two classes (normal and big) of healthy-looking primary spermatocytes. In such case, different classes of spermatocytes most probably originated from diploid and tetraploid SSCs. Such spermatocytes gave rise to haploid and diploid spermatozoa observed in some individuals. However, the presence of single big primary spermatocytes in the cyst containing normal size cells cannot be explained in that way, since all cells in one cyst are descendants of a single SSC. The possible mechanism for formation of bigger spermatocytes may be cell fusion, which was reported during formation of unreduced gametes in the gynogenetic hybrid fish (Wang et al., 2016). On the other hand, the presence of different cell lines (but of the same ploidy) within the same cyst was described in *P. ridibundus* and *P. lessonae* (Haczkiewicz et al., 2017).

Assuming that genome elimination and endoreplication in gonocytes were correct, the resulting spermatocytes should have the same size as spermatocytes of the parental species. We have indeed obtained such results for a control *P. ridibundus*, whereas in all hybrids (RL, RLL, RRL) we observed two classes of primary spermatocytes, i.e., normal and big. The big spermatocytes most probably reflected 8n cells that would give rise to diploid sperm. The sizes of regular spermatocyte nuclei seem to be correct when we assume that elimination of genomes takes place in G cells, while SSCs and their descendant spermatocytes have 2n and 4n genomes, respectively. If RRL eliminates L it will result in RR spermatocytes; RLL eliminates R and results in LL spermatocytes; and RL eliminates L (preferably) or R, and—after duplication - restore LL or RR diploid chromosomal set. In this section we measured nuclei, not whole cells, and RR spermatocyte nuclei of RRL frog had the biggest DNA values and the biggest nuclei (9.88 µm), LL spermatocyte nuclei of RLL frog had the lowest DNA value and the smallest nuclei (9.26 µm), whereas RL had intermediate values of DNA and eliminate R or L, so the mean nuclei size is also intermediate (9.40 µm). The differences between normal-sized spermatocytes containing L (in RLL) and R (in RRL) genomes most probably reflects the higher DNA content in *P. ridibundus* as compared to *P. lessonae* (Vinogradov et al., 1990; Ogielska et al., 2005).

The gonocyte sizes were measured in the parental species and were 15.73 µm in *P. lessonae* and 17.74 µm in *P. ridibundus* (Haczkiewicz et al., 2017) and their sizes corresponded well to DNA content which is higher in *P. ridibundus* (Vinogradov et al., 1990; Ogielska et al., 2005). In this study we dealt with hybrid individuals and cell sizes in diploid RL tadpoles were the smallest (16.39 μm), slightly larger in RRL (17.44 μm) and the largest in RLL (18.29 μm), which is not in full agreement with DNA content. However, cells are generally bigger in *P. lessonae* than in *P. ridibundus* despite the amount of DNA. Such cell size values were obtained for erythrocytes when their long axes were measured: 24.9 µm in *P. lessonae,* 23.37 µm in *P. ridibundus* and respective values for *P. esculentus*: 24.2 µm in RL, 29.5 µm in RRL and 30.33 µm in RLL (Kierzkowski et al., 2011).

Various sizes of germline cells were also found in the north American salamander *Ambystoma-laterale-jeffersonianum-texanum-tigrinum* complex (LJTTi), in which two allotriploid all-female taxa reproduce by gynogenesis. In newly metamorphosed hybrids *A. platineum* (JJL) and *A. tremblayi* (JLL), ovaries contained two sizes of zygotene/pachytene oocytes: only those that were at least twice the size of the others were 3n and would give rise to functional ova (Sessions, 1982).

### Crossing experiments with hybrid males evidence that only some types of genomic compositions in spermatozoa give viable progeny

The fertilization success of hybridogenetic male *P. esculentus* is clearly lower than that of both parental species *P. ridibundus* and *P. lessonae* (Günther, 1990; Berger and Rybacki, 1992; Berger and Rybacki, 1994). Motile spermatozoa did not differ in tail/head length ratio and velocity between hybrids and parental species, but the hybrids produced less spermatozoa (Reyer et al., 2003) and/or produced deficient ones (this study). Not only the density of sperm, but also proper chromosome content affects the fertilization success in hybrids and the mortality rate of the offspring (Dedukh et al., 2022b).

Variability of the chromosomal composition of SSCs was much higher than that of spermatozoa (Figure 12, Table 1). It suggests that during spermatogenesis, a large portion of abnormal SSCs degenerated and did not transform into functional gametes. Such massive degeneration of SSCs was confirmed by the analysis of histological sections of adult males. The spermatozoa produced by diploid and triploid males also varied in genomic composition. The greatest gamete diversity was observed in diploid RL males, in which we found not only dominating haploid R, but also RR, L and RRRR spermatozoa. Similarly, the predominant formation of R spermatozoa by diploid RL males was noticed for L-E systems (Dedukh et al., 2019). Our study shows that in RLL males, L gametes predominated, and R gametes had low frequency. Triploid RRL males usually produced haploid R sperm but also diploid RR sperm. Not all abnormal germ cells degenerated, as evidenced by the presence of aneuploid spermatozoa accounting for significant fractions in RL and RLL, 22% and 18.48% respectively, and small group of 4.38% in RRL (Figure 9). Histological observations confirmed degeneration of some spermatozoa, which suggest that not all aneuploid gametes will survive until mating and fertilization. In fish hybrid males evolving from crosses of different *Danio* species ploidy disorders were also recorded from below 1C to 2C DNA content an in rare cases diploid sperms gave rise to triploid progeny (Endoh et al., 2020). For such a phenomenon as the production of several types of sperm by one male, Vinogradov et al. (1991) proposed the term “hybrid amphispermy”. A similar phenomenon was observed by Pruvost et al. (2013), Biriuk et al. (2016), Doležálková-Kaštánková and Mazepa (2021), Svinin et al. (2021), and most recently by Pustovalova et al. (2022).

## Data Availability

The original contributions presented in the study are included in the article/Supplementary Material, further inquiries can be directed to the corresponding author.
