# Supplementary material for: Genome elimination from the germline cells in diploid and triploid male water frogs Pelophylax esculentus
Source: Front Cell Dev Biol. 2022 Oct 14;10:1008506. doi: 10.3389/fcell.2022.1008506 (PMC9615423; doi:10.3389/fcell.2022.1008506)
Supplement: Supplementary file 1 [file DataSheet2.docx]

**Supplementary Information 1**

**Title:**

**Genome elimination from the germline cells in diploid and triploid male water frogs *Pelophylax* *esculentus***

**Authors:**

Magdalena Chmielewska^1^†*, Mikołaj Kaźmierczak^1, 2^†, Beata Rozenblut-Kościsty^1^, Krzysztof Kolenda^1^, Anna Dudzik^1^, Dmitrij Dedukh^3^, Maria Ogielska^1^

*Corresponding author: Magdalena Chmielewska [magdalena.chmielewska@uwr.edu.pl](mailto:magdalena.chmielewska@uwr.edu.pl)

**
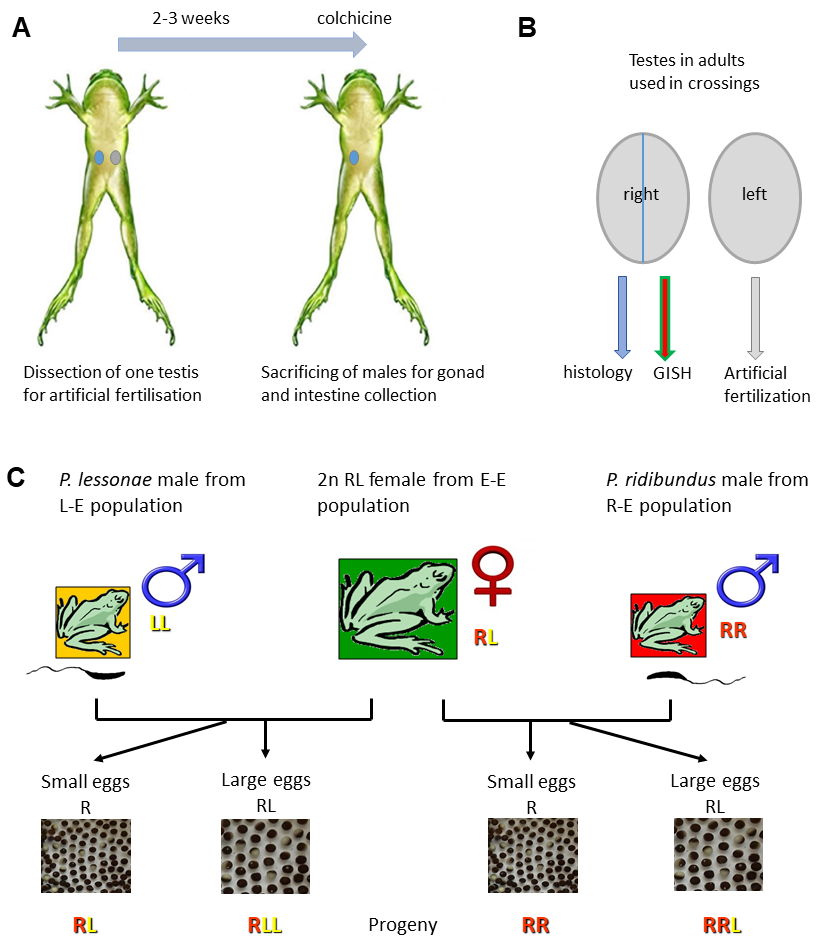
**

**Supplementary Figure 1. Experimental design of the study.** (A, B) Workflow of male fertility and genome transmission study. (A) Males were first taking part in *in vitro* crossings, then, after 2-3 weeks they were injected with colchicine and 24 hour later were sacrificed. (B) Left testis was used for crossing, right testis was cut in halves and fixed for histology and chromosomal preparation. (C) Schematic view of the in vitro crossing design to obtain progeny of different genomic make up including diploid RL and triploid RLL and RRL individuals.

**
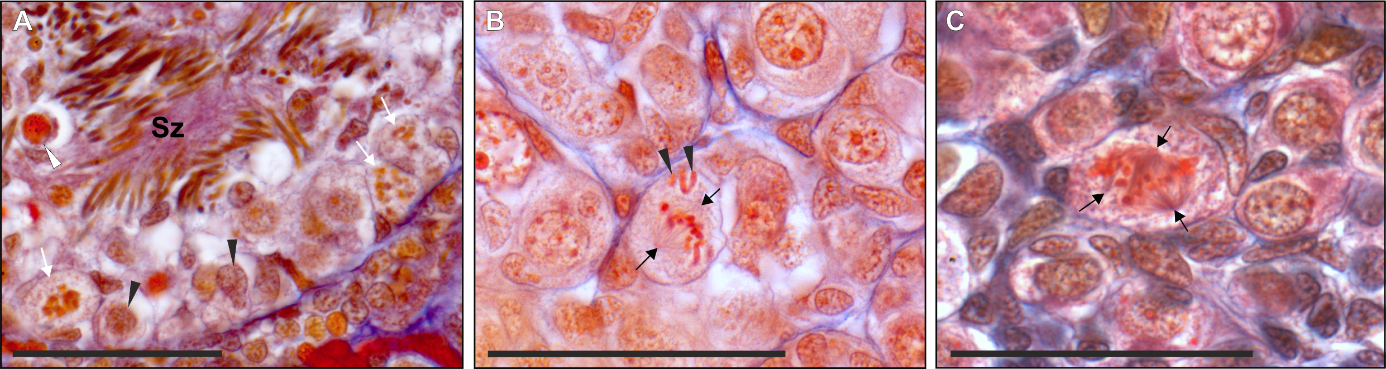
**

**Supplementary Figure 2. Mitotic abnormalities in male gonads.** (A) Normal mitosis in the SSCs of an adult male no. 5 after blocking of mictotubule polymerization by colchicine (black arrows), note normal (black arrowheads) and degenerating SSC (white arrowhead), Sz - spermatozoa. (B) Misaligned chromosomes (black arrowheads) in the mitotic metaphase of the gonocyte in the RL tadpole at G. st. 44, at the left side of the chromosomal plate one pole of the mitotic spindle is visible. (C) Multipolar mitosis, note the 3 spindle poles (white arrows) and metaphase chromosomes organized in several planes. Scale bar 50 µm.

**
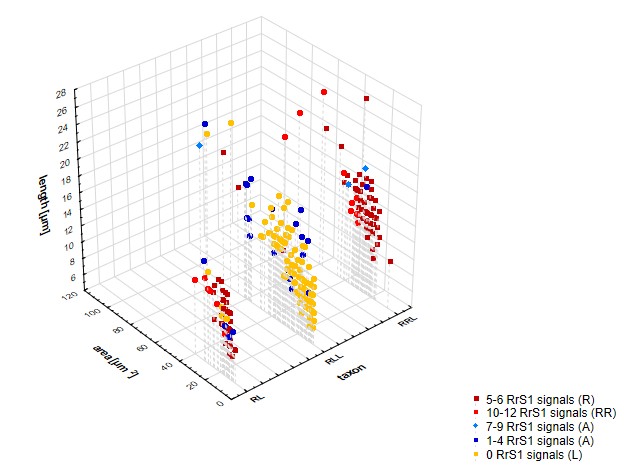
**

**Supplementary Figure 3. Distribution of sperm size and types in diploid RL and triploid RLL and RRL males according to the number of centromeric RrS1 signals**. Measurements of the sperm heads length and area was done in digital images of chromosomal preparations probed with GISH/FISH method with the centromeric RrS1 probe. Several classes of spermatozoa were distinguished based on the number of centromeric signals: (R) haploid set of P. ridibundus signals, (RR) diploid set of *P. ridibundus* signals, (L) spermatozoa without RrS1 signals may contain haploid (L) or diploid (LL) *P.* *lessonae* chromosome sets, (A) Spermatozoa with aneuploid number of RrS1 *P.* *ridibundus* signals.
